# Fluorescent BAPAD Dendrimeric Antigens Are Efficiently Internalized by Human Dendritic Cells

**DOI:** 10.3390/polym8040111

**Published:** 2016-03-26

**Authors:** Pablo Mesa-Antunez, Daniel Collado, Yolanda Vida, Francisco Najera, Tahia Fernandez, Maria Jose Torres, Ezequiel Perez-Inestrosa

**Affiliations:** 1 Department of Organic Chemistry, University of Malaga, IBIMA, 29071 Malaga, Spain; pmantunez@uma.es (P.M.-A.); dcollado@uma.es (D.C.); yolvida@uma.es (Y.V.); najera@uma.es (F.N.); 2 Andalusian Centre for Nanomedicine and Biotechnology- BIONAND, Parque Tecnologico de Andalucia, 29590 Malaga, Spain; 3 Research Laboratory, Regional University Hospital of Malaga-IBIMA, 29010 Malaga, Spain; tahiadfd@gmail.com (T.F.); mjtorresj@gmail.com (M.J.T.); 4 Allergy Service, Regional University Hospital of Malaga-IBIMA, 29010 Malaga, Spain

**Keywords:** dendrimeric antigen, naphthalimide, fluorescence, flow cytometry, confocal microscopy, amoxicillin, allergy

## Abstract

A new fluorescent dendrimeric antigen (DeAn) based on a dendron with amoxicilloyl terminal groups was synthesized. The synthesis was carried out using a novel class of all-aliphatic polyamide dendrimer (BisAminoalkylPolyAmide Dendrimers, or BAPAD) involving the direct condensation of 3,3′-diazidopivalic acid as a building block. Iterative azide reduction/amide formation increases the dendrimer generation. The BAPAD dendrimer was designed with a cystamine core. Reduction of the disulfide bond allows the incorporation of BAPAD dendrons into a 1,8-naphthalimide functionalized with a maleimide group. The fluorescence properties of DeAn were studied in PBS and compared with the properties of an equivalent dendron possessing amino-terminal groups. Both molecules shown high fluorescence quantum yields in PBS and could readily be visualized by fluorescence microscopy. DeAn was used as a synthetic antigen in a biomedical assay that tests their potential as an amoxicillin carrier in drug internalization by dendritic cells (DC) from tolerant and allergic patients. Cytometry data suggest that the dendrons are non-toxic and easily internalized by DCs, while confocal microscopy images indicate that the compounds are preferentially accumulated in the cytoplasm. These results indicate that BAPAD dendrons are good candidates for synthetic scaffolds for biomedical applications.

## 1. Introduction

Since their conception, dendrimers have drawn considerable interest due to their potential applications in many fields of science. The body of literature concerning dendrimers continues to grow rapidly, showing the appeal that these polymeric compounds still have [1,2,3]. Recently, we presented the development of a simple synthetic approach for a novel class of all-aliphatic polyamide dendrimer (BisAminoalkylPolyAmide Dendrimer or BAPAD) consisting of the direct condensation of 3,3′-diazidopivalic acid as a building block. This structure uses carboxylic acid groups to connect each monomeric subunit via amide linkages, while the presence of azide groups in the form of “protected amines” (pro-amines) allows dendrimer growth to be controlled at each generation [4]. This strategy represents a facile synthetic method for aliphatic polyamide dendrimers based on a divergent approach. While azido-terminal dendrimers of each generation are soluble in common organic solvents, equivalent amino-terminal dendrimers are soluble in aqueous media. These terminal amino groups represent a potentially interesting route for introducing biomolecules of interest. Commercial dendrimers like PAMAM (polyamidoamine) or PPI (polypropylenimine) have been subjected to functionalization of these terminal groups [5,6,7]. PAMAM dendrimers are widely used as scaffolds for biomedical applications. The bio-safety of these dendrimers has been widely studied [8]. Our main interest when working with these polymeric structures has been related to their use as dendrimeric antigens (DeAns) in drug allergy studies. In an allergic reaction to medicines, the pharmaceutical agent usually induces a response by acting as a hapten. A hapten is a small molecule or defined chemical structure that is unable to induce the allergic response by itself. To induce allergy, these haptens need to be conjugated to a carrier protein or macromolecule in order to be recognized by the immunological system [9,10]. In the case of DeAns, the role of the carrier protein is performed by the dendrimer and the conjugate is established by means of a covalent bond. We have focused mainly on the preparation and characterization of antigenic determinant of betalactamic antibiotics: cephalosporins [11,12] and penicillin [13,14]. We have been working on expanding the all-aliphatic polyamide BAPAD dendrimer scaffold structures for the synthesis of new DeAns. Moreover, using a central core of a dendrimer susceptible to cleavage and reassembly under certain conditions expands the possibilities of these structures for linking different molecules or surfaces. Through a reversible reduction/oxidation process, a disulfide bond present in proteins and other organic compounds can be broken to obtain thiol groups that can be coupled to appropriate structures [15,16,17] or surfaces [18]. We have designed BAPAD dendrimers with a cystamine core and have already demonstrated that it is possible to cleavage the disulfide bond of these new BAPAD dendrimers. The resulting dendrons present a thiol group on its focal point and have interesting properties for biomedical applications when supported over gold surfaces and used as a nanoplasmonic biosensor [19]. We have designed and developed a complete biosensing strategy by combining specifically synthesized dendron-based amoxicillin conjugates with nanostructured gold surfaces leading to a fast, reliable, highly sensitive, and label-free analysis. However, we also propose using the new BAPAD dendron for other studies related to amoxicillin allergy. For instance, we are interested in the study of dendritic cells activation by DeAns presenting the antigenic determinant of amoxicillin (AXO group) in the surface.

**Figure 1:**
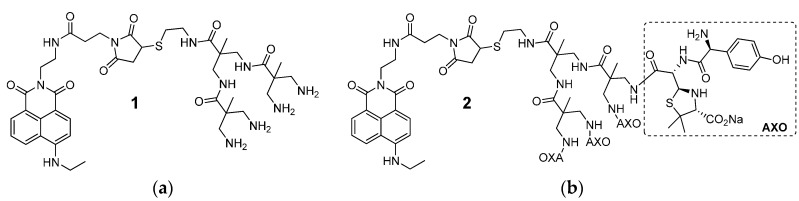
Second-generation BAPAD fluorescent dendrons: (**a**) with terminal amino groups; (**b**) with terminal amoxicillin groups.

In this work we present the synthesis of two new fluorescent dendrons obtained by a Michael addition of the second generation BAPAD dendron with a thiol group on its focal point to a maleimide ring attached to a naphthalimide derivative through a small spacer. The free amino (ammonium in water) groups (Figure 1A, AMO) on the surface of the dendrimeric structures allow their haptenization with amoxicillin (Figure 1B, AXO).

## 2. Materials and Methods

### 2.1. Materials

3,3’-Dichloropivalic acid, triphenylphosphine, ethylamine, 4 M HCl/dioxane and dithiothreitol were purchased from Sigma-Aldrich, Europe (Taufkirchen, Germany). Cystamine dihydrochloride was purchased from Alfa-Aesar (Karlsruhe, Germany), β-Alanine from Fluka (Taufkirchen, Germany) and amoxicillin sodium salt from GlaxoSmithKline (Madrid, Spain). Solvents were obtained from Sigma-Aldrich and Merck (Madrid, Spain), and they were used as received without further purification unless stated. Compounds **8** and **10** were prepared according to a previously published synthetic procedure [20,21].

### 2.2. Structural Characterization

^1^H NMR and ^13^C NMR spectra were recorded using Bruker ARX-400 (Bruker, Billerica, MA, USA) at 400 MHz for ^1^H and 100.6 MHz for ^13^C, Bruker Biospin Avance III 400 at 400 MHz for ^1^H and 100.6 MHz for ^13^C, Bruker Ascend™-400 at 400 MHz for ^1^H and 100.6 MHz for ^13^C or in a Bruker AVANCE™-600 AT 600 MHz for ^1^H and 150 MHz for ^13^C spectrometers. Mass spectra were performed in a mass spectrometer with electronic impact ionization (70 eV) Thermo Scientific DSQ II Single Quadrupole GC/MS with Focus GC. The HRMS (Electrospray Ionization Time of Flight, ESI-TOF) mass spectra (MS) were performed on a High Resolution Mass Spectrometer Orbitrap, Q-Exactive (Thermo Fisher Scientific, Waltham, MA, USA) Electrospray Ionization (H-ESI-II), coupled to a Liquid Chromatographer uHPLC Ultimate 2000 Dionex (Thermo Fisher Scientific, Waltham, MA, USA). Matrix-Assisted Laser Desorption/Ionization (MALDI) experiments were performed on a 4700 Proteomics Analyzer MALDI-TOF mass spectrometer. UV–Vis absorption spectra were registered on a Hewlett-Packard 8452A Diode Array spectrophotometer and an AGILENT 8453 spectrophotometer (Santa Clara, CA, USA).

### 2.3. General Procedure for Amide Formation

A mixture of the corresponding amine, CH_2_Cl_2_ and 10% aqueous sodium hydroxide was cooled to 0 °C. 3,3’-Diazidopivalic acid chloride (**3**) freshly prepared in CH_2_Cl_2_ (see Figures S5 and S6) was added dropwise while the temperature was kept at 0 °C. After addition, the reaction was allowed to reach room temperature and stirred overnight. The organic layer was then separated, washed with 10% aqueous sodium hydroxide, 1 M aqueous HCl, and water, dried over MgSO_4_ and the solvent removed to obtain generation one dendrimer **4** as a solid. Yield: 83% ^1^H NMR (CDCl_3_, 400 MHz; Figure S7) δ 6.84 (t, *J* = 5.3 Hz, 2H, N***H***C_2_H_4_), 3.57 (dd, *J* = 11.6, 5.5 Hz, 4H, NHC***H*_2_**CH_2_S), 3.53 (d, *J* = 12.3 Hz, 4H, N_3_C***H***H), 3.47 (d, *J* = 12.3 Hz, 4H, N_3_CH***H***), 2.82 (t, *J* = 6.5 Hz, 4H, NHCH_2_C***H*_2_**S), 1.24 (s, 6H, C***H*_3_**). ^13^C NMR (CDCl_3_, 101 MHz; Figure S8) δ 173.10, 56.07, 47.28, 38.73, 37.50, 19.10. HRMS (ESI-TOF; Figure S23) calcd. for C_14_H_25_N_14_O_2_S_2_ [M + H]^+^ 485.17208, found 485.17308. Purification by silica gel column chromatography was only necessary for the second-generation dendrimer, **6**, and gave the desired compound as a syrup. Yield: 67% *R_f_* = 0.32 (EtOAc:Hexane 1:1 to 2:1) ^1^H NMR (CDCl_3_, 400 MHz; Figure S11) δ 7.78 (t, *J* = 5.7 Hz, 2H, N***H***CH_2_CH_2_S), 7.63–7.57 (m, 4H, N***H***CH_2_), 3.62–3.44 (m, 24H, NHC***H*_2_**), 3.32 (dd, *J* = 14.1, 5.4 Hz, 4H, NHC***H*_2_**CH_2_S), 2.84 (t, *J* = 6.5 Hz, 4H, NHCH_2_C***H*_2_**S), 1.28 (s, *J* = 7.8 Hz, 12H, C***H*_3_***^G^*^2^), 1.16 (s, 6H, C***H*_3_***^G^*^1^). ^13^C NMR (CDCl_3_, 101 MHz; Figure S12) δ 175.20, 174.14, 56.02, 55.99, 48.17, 47.58, 42.47, 38.95, 38.08, 29.60, 20.07, 19.00. MS (ESI; Figure S25) calcd. for C_34_H_57_N_30_O_6_S_2_ [M + H]^+^ 1045.45, found 1045.31.

### 2.4. General Procedure for Azide Reduction

The corresponding azide derivative was dissolved in THF and cooled to 0 °C. A solution in THF of PPh_3_ was then added. After addition, the reaction was allowed to reach room temperature and then heated to 60 °C overnight. Water was then added and the reaction heated to 70 °C for 3–4 h. After this time, the solvent was removed, 1 M aqueous HCl added, and the aqueous phase washed with CH_2_Cl_2_. After freeze-drying the desired compounds were obtained as solids. Generation one, **5**. Yield: 96%. ^1^H NMR (D_2_O, 400 MHz; Figure S9) δ 3.48 (t, *J* = 6.5 Hz, 4H, NHC***H*_2_**CH_2_S), 3.30 (d, *J* = 13.6 Hz, 4H, N_3_C***H***H), 3.09 (d, *J* = 13.5 Hz, 4H, N_3_CH***H***), 2.79 (t, *J* = 6.5 Hz, 4H, NHCH_2_C***H*_2_**S), 1.36 (s, 6H, C***H*_3_**). ^13^C NMR (DMSO, 101 MHz; Figure S10) δ 171.80, 43.44, 42.52, 38.72, 36.52, 19.07. HRMS (ESI-TOF; Figure S24) calcd. for C_14_H_33_N_6_O_2_S_2_ [M + H]^+^ 381.21009, found 381.21019. Generation two, **7**. Yield: 92%. ^1^H NMR (D_2_O, 400 MHz; Figure S13) δ 3.52–3.14 (m, 28H, NHC***H*_2_**CH_2_S, NHC***H*_2_**), 2.82 (t, *J* = 6.5 Hz, 4H, NHCH_2_C***H*_2_**S), 1.45 (s, 12H, C***H*_3_***^G^*^2^), 1.16 (s, 6H, C***H*_3_***^G^*^1^). ^13^C NMR (CDCl_3_, 101 MHz; Figure S14) δ 176.33, 173.22, 47.00, 44.33, 44.17, 43.94, 43.63, 43.62, 38.57, 36.27, 18.18, 17.35. MS (MALDI-TOF; Figure S26) calcd. for C_34_H_72_N_14_O_6_S_2_ [M + H]^+^ 837.5278, found 837.5259.

### 2.5. Synthesis of Compound **9**

In a round-bottom flask **8** (200 mg, 0.48 mmol) and ethylamine (43 mg, 70 wt % in water, 0.96 mmol) were dissolved in 3 mL of dimethyl sulfoxide and heated to 60 °C during 12 h while stirring. After completion, 100 mL of dichloromethane were added and the mixture washed with water, 1 M aqueous HCl and water again, and dried with MgSO_4_. After filtration the solvent was removed to give compound as a solid. To this solid (233 mg, 0.57 mmol) dissolved in tetrahydrofuran, 4 M HCl in dioxane in a 1:1 proportion was added and the mixture stirred during 4 h. After completion the solvent was removed and the crude resuspended in diethyl ether at 4 °C. After precipitation the solvent was removed to give **9** as a solid. Yield: 41% (two steps). ^1^H NMR (D_2_O, 400 MHz; Figure S15) δ 7.53 (d, *J* = 7.2 Hz, 1H, ***H*_5_**), 7.43 (d, *J* = 8.2 Hz, 1H, ***H*_7_**), 7.32 (d, *J* = 8.6 Hz, 1H, ***H*_2_**), 6.91 (t, *J* = 7.8 Hz, 1H, ***H*_6_**), 6.01 (d, *J* = 8.8 Hz, 1H, ***H*_3_**), 3.96 (t, *J* = 6.1 Hz, 2H, ***H*_11_**), 3.19–3.00 (m, 4H, ***H*_9_**, ***H*_12_**), 1.22 (t, *J* = 7.2 Hz, 3H, ***H*_10_**).^13^C NMR (CDCl_3_, 101 MHz; Figure S16) δ 164.98, 163.68, 151.10, 134.25, 130.56, 128.22, 128.03, 123.41, 118.66, 118.29, 104.56, 103.79, 38.15, 37.80, 37.38, 13.11. MS (ESI; Figure S27) calcd. for C_16_H_18_N_3_O_2_ [M + H] 284.14, found 284.52.

### 2.6. Synthesis of Compound **11**

To **9** (60 mg, 0.2 mmol) in 4 mL of dimethylformamide, **10** (64 mg, 0.24 mmol) and DIPEA (0.17 mL, 1 mmol) were added and the mixture was stirred during 12 h. After completion, the solvent was removed and the solid washed with methanol and resuspended in diethyl ether at 4 °C. After precipitation the solvent was removed to give **11** as a solid. Yield: 52%. ^1^H NMR (DMSO, 400 MHz; Figure S17) δ 8.70 (d, *J* = 8.5 Hz, 1H, ***H*****_5_**), 8.43 (d, *J* = 7.3 Hz, 1H, ***H*_7_**), 8.27 (d, *J* = 8.3 Hz, 1H, ***H*_2_**), 8.06 (bs, 1H, N***H***CO), 7.69 (m, 2H, ***H*_6_**, N***H***CH_2_CH_3_), 6.98 (s, 2H, ***H***C=C***H***), 6.77 (d, *J* = 8.6 Hz, 1H, ***H*_3_**), 4.07 (t, *J* = 5.3 Hz, 2H, ***H*_11_**), 3.55 (t, *J* = 7.3 Hz, 2H, ***H*_14_**), 3.49–3.38 (m, 2H, ***H*_9_**), 2.24 (t, *J* = 7.5 Hz, 2H, ***H*_15_**), 1.31 (t, *J* = 7.0 Hz, 3H, ***H*_10_**). ^13^C NMR (DMSO, 101 MHz; Figure S18) δ 170.74, 169.45, 164.02, 163.14, 150.45, 134.51, 134.24, 130.58, 129.57, 128.49, 124.17, 122.04, 120.10, 107.75, 103.66, 37.53, 36.66, 34.01, 33.86, 13.67. MS (ESI; Figure S28) calcd. for C_23_H_23_N_4_O_5_ [M + H] 435.17, found 435.51.

### 2.7. Synthesis of Compound **1**

DTT (6.30 mg, 4.08 × 10^−2^ mmol) was added to an Ar-filled flask with **7** (11.6 mg, 10.2 × 10^−3^ mmol) dissolved in 2 mL of degasified water and the mixture was stirred for 24 h at RT. After that, it was washed four times with degasified dichloromethane and **11** (9 mg, 2.04 × 10^−2^ mmol) dissolved in degasified dimethyl sulfoxide was added and stirred for 24 h at room temperature. After completion the solvent was removed under a high vacuum to give **1** as a solid. Yield: 83%. ^1^H NMR (D_2_O, 400 MHz; Figure S1) δ 7.74 (m, 2H, ***H*_5_**, ***H*_7_**), 7.55 (d, *J* = 8.7 Hz, 1H, ***H*_2_**), 7.09 (t, *J* = 7.9 Hz, 1H, ***H*_6_**), 6.20 (d, *J* = 8.9 Hz, 1H, ***H*_3_**), 3.94–2.59 (m, 27H, NHC***H*_2_**CH_2_S, NHC***H*_2_**, NC***H*_2_**CH_2_CO, NCH_2_C***H*_2_**CO, NHCH_2_C***H*_2_**N, SC***H***CO, SCHC***H*_2_**CO) 2.25–2.14 (m, 2H, NHCH_2_C***H*_2_**S), 1.25 (s, 6H, C***H*_3_***^G^*^2^), 1.14 (t, *J* = 7.2 Hz, 3H, NHCH_2_C***H*_3_**), 0.93 (s, 3H, C***H*_3_***^G^*^1^). ^13^C NMR (CDCl_3_, 101 MHz; Figure S2) δ 178.53, 177.61, 176.04, 173.23, 172.86, 165.69, 164.65, 151.42, 135.04, 131.26, 128.87, 128.46, 124.17, 120.05, 119.25, 105.91, 104.24, 46.94, 44.26, 44.08, 43.52, 39.03, 38.38, 37.91, 35.39, 33.20, 30.24, 29.99, 18.00, 17.89, 17.25, 13.13. MS (MALDI-TOF; Figure S21) calcd. for C_40_H_60_N_11_O_8_S [M + H]^+^ 854.4347, found 854.4686.

### 2.8. Synthesis of Compound **13**

To a mixture of **7** (50 mg, 0.044 mmol) in 1 mL of water, Na_2_CO_3_·10H_2_O was added to a pH = 10. Then amoxicillin as sodium salt (273 mg, 0.704 mmol, in four portions) was added. Five milligrams of fresh amoxicillin were added every 12 h for 48 h and the resulting mixture purified by gel filtration chromatography (Sephadex G-10, water) to obtain **13** as a solid. Yield: 72%. ^1^H NMR (D_2_O, 400 MHz; Figure S19) δ 6.98 (d, *J* = 7.4 Hz, 16H, ***H*_12*’*_**), 6.48 (d, *J* = 6.9 Hz, 16H, ***H*_11*’*_**), 5.37–4.75 (m, 8H, ***H*_5*’*_**), 4.42–4.09 (m, 16H, ***H*_6*’*_**, ***H*_9*’*_**), 3.50–2.97 (m, 36H, ***H*_3*’*_**, NHC***H*_2_**, NHC***H*_2_**CH_2_S), 2.72 (s, 4H, NHCH_2_C***H*_2_**S), 1.56–0.86 (m, 66H, C***H*_3_**). ^13^C NMR (CDCl_3_, 10 MHz; Figure S20) δ 174.95, 174.37, 172.29, 170.65, 157.00, 129.82, 129.51, 116.95, 116.43, 116.37, 116.30, 75.63, 74.76, 66.61, 65.05, 59.74, 58.54, 56.82, 56.58, 55.11, 27.90, 27.22, 26.86, 26.74. MS (MALDI-TOF; Figure S29) calcd. for C_162_H_225_N_38_O_46_S_10_ [M + H]^+^ 3758.3642, found 3757.9983.

### 2.9. Synthesis of Compound **2**

Following the procedure to obtain compound **1** but using compound **13** (20 mg, 5.1·10^−3^ mmol) as dendrimer, generated compound **2** as a solid. Yield: 81%. ^1^H NMR (DMSO, 600 MHz; Figure S3) δ 8.68 (t, *J* = 10.1 Hz, 1H, ***H*_5_**), 8.41 (t, *J* = 7.0 Hz, 1H, ***H*_7_**), 8.25 (t, *J* = 8.4 Hz, 1H, ***H*_2_**), 8.09 (s, 1H, N***H***CH_2_CH_3_), 7.79–7.59 (m, 2H, ***H*_6_**, N***H***CO), 7.40–7.10 (m, 8H, ***H*_12*’*_**), 6.83–6.58 (m, 9H, C_3_***H***, ***H*_11*’*_**), 5.12–4.51 (m, 4H, ***H*_5*’*_**), 4.37–3.89 (m, 4H, ***H*_6*’*_**), 3.73–2.63 (m, 35H, ***H*_3*’*_**, ***H*_9*’*_**, NHC***H*_2_**CH_2_S, NHC***H*_2_**, NC***H*_2_**CH_2_CO, NCH_2_C***H*_2_**CO, NHCH_2_C***H*_2_**N, SC***H***CO, SCHC***H*_2_**CO), 2.26–2.12 (m, 2H, NHCH_2_C***H*_2_**S), 1.58–0.79 (m, 36H, C***H*_3_**). ^13^C NMR (DMSO, 151 MHz; Figure S4) δ 206.48, 177.42, 176.57, 174.84, 169.31, 164.03, 163.16, 150.44, 134.20, 130.54, 129.58, 128.46, 124.16, 122.01, 120.08, 115.08, 107.73, 103.64, 37.52, 36.74, 34.85, 33.01, 30.65, 27.99, 13.65. MS (MALDI-TOF; Figure S22) calcd. for C_104_H_136_N_23_O_28_S_5_ [M + H]^+^ 2314.8528, found 2314.8547.

### 2.10. Fluorescence Characterization

The fluorescence emission spectra were recorded on a Jasco FP-750 spectrofluorometer (Tokyo, Japan) and a FLS920 Edinburgh Instruments spectrofluorometer (West Lothian, UK). Flow cytometry measurements were performed using a FACSCantoII (Becton–Dickinson, Franklin Lakes, NJ, USA) instrument with 488 and 633 nm lasers and FACSDiva software (Erembodegem, Belgium). Confocal images were taken with a Leica Confocal SP5 II Multispectral microscope (Wetzlar, Germany).

### 2.11. Molecular Dynamics Simulations

Full details of the Dendron Building and Molecular Dynamics Simulations are described in the Supplementary Materials. Briefly, initial dendron conformations were generated using the Dendrimer Building Tool (DBT) [22]. Simulations were performed using the AMBER12 MD software package [23]. We utilized the AMBER force field parameters (parm99) and those not described were transferred from the General Atom Force Field (GAFF) parameters [24].

The system was minimized and then heated to 300 K over 40 ps. Simulations were run, using water as explicit solvent, in NPT ensemble at 300 K and 1 atm for 40 ns. Non-bonded interactions were cutoff at 9 Å. Time steps of 2 fs were taken with implementation of the SHAKE routine [25]. Dendrons were equilibrated for 2 ns and starting from these configurations, production runs of 20 ns trajectories were performed under an NPT ensemble. Trajectory analyses were performed using the Amber modules *ptraj* and *cpptraj.* Snapshots from the trajectories within this paper were created with VMD software [26].

### 2.12. Generation of Monocyte-Derived DCs

Fresh peripheral blood mononuclear cells (PBMCs) obtained from 40 mL of each individual were used for monocytes purification by means of anti-CD14 microbeads following the manufacturer’s protocol (Miltenyi Biotec, Bergisch Gladbach, Germany). To generate DCs, monocytes (CD14+ cells) were incubated in complete medium (CM) containing Roswell Park Memorial Institute 1640 medium (Life Technologies, Invitrogen, Carlsbad, CA, USA) supplemented with 10% Fetal Calf Serum (FCS; Life Technologies, Carlsbad, CA, USA), streptomycin (100 µg·mL^−1^), gentamicin (1.25 U·mL^−1^) as well as recombinant human rhGM-CSF (200 ng·mL^−1^) and rhIL-4 (100 ng·mL^−1^) (both from R & D Systems Inc., Minneapolis, MN, USA) for 5 days at 37 °C and 5% CO_2_. The resulting DCs were then recovered and used in the experiments.

The study was conducted according to the declaration of Helsinki and all patients and controls participating in the study gave their informed consent and protocols were approved by institutional ethical committees (Ethical Committee of Malaga).

### 2.13. Flow Cytometry-Based Detection of Compounds **1** and **2** in DCs

DCs were incubated at 1 × 10^5^ cells/well in 96-well plates (Nunc, Roskilde, Denmark) with compounds 1 and 2 at 10, 1, and 0.1 µM in CM for 3 h, 24 h, and 48 h at 37 °C. Cells were then analyzed using a FACSCanto II flow cytometer (BD Biosciences, San Diego, CA, USA) and the data were processed with FLOWJO software (Tree Star, Inc., Ashland, OH, USA).

### 2.14. Cell Toxicity Analyses

The determination of the cytotoxic effects of compounds 1 and 2 on DCs was performed by flow cytometry. After incubation, cells were stained with Live/Dead NearIR (Life Technologies-Invitrogen, Waltham, MA, USA) for 15–20 min. Cells were then acquired in a flow cytometer (FACSCanto II flow cytometer, BD Biosciences, San Diego, CA, USA) Data were after analyzed using FLOWJO software (Tree Star, Inc., Ashland, OH, USA). The cytotoxicity of NCs on DCs was expressed as a percentage of live cells.

## 3. Results and Discussion

### 3.1. Synthesis

**Scheme 1:**
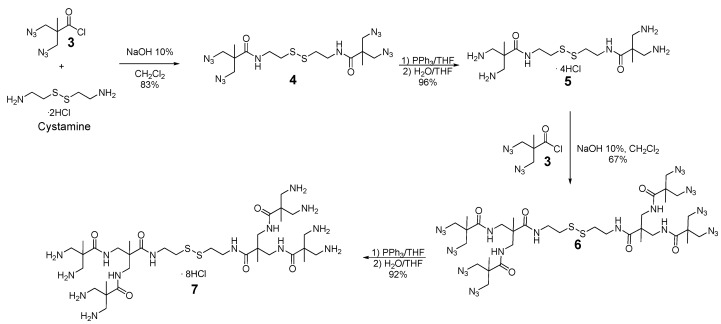
Synthesis of generation 2 dendrimer **7**, by an iterative sequence of coupling and deprotection–reduction.

The first synthetic procedure described for BAPAD dendrimer synthesis used an iterative process of amine deprotection/amide formation via a divergent strategy. However, such a divergent strategy implies the reduction of azido groups in the presence of the chromophoric core. To avoid this potentially problematic reaction [27], here we have instead used a convergent synthetic procedure. In this approach the synthesis of the dendrimeric and chromophore structures are carried out first before being coupled together. Disulfide bonds can be subjected to a reversible sequence of disassembly/assembly, making them suitable for this synthetic procedure [16,28]. A cystamine core was used for the dendrimeric structure to prove the required disulfide bond that can be cleavage under reductive conditions. Scheme 1 shows the iterative synthetic pathway used to obtain the target compound.

First amide formation between cystamine dihydrochloride and **3** was carried out in NaOH/CH_2_Cl_2_ under Schotten-Baumann conditions [29,30] to obtain generation 1 dendrimer After a Staudinger reduction [31,32] of the azide groups with PPh_3_ in THF to provide the ammonium derivative, a second sequence of coupling and reduction led to generation 2 dendrimer, **7**.

**Figure 2:**
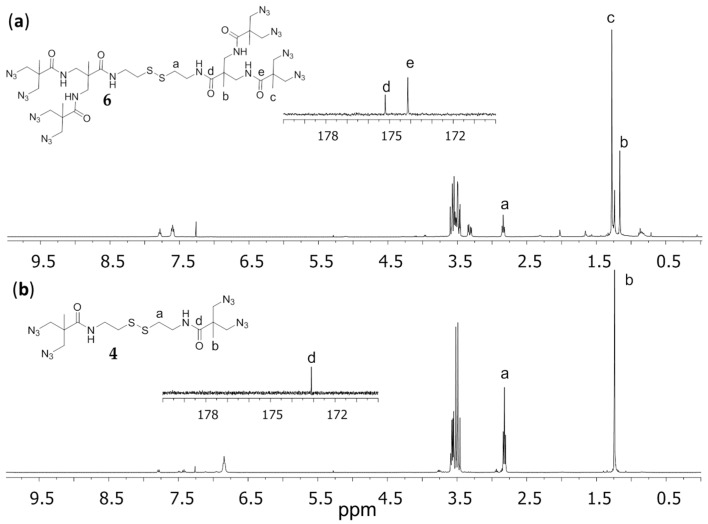
NMR comparison between: (**a**) generation 2 azide derivative **6** and (**b**) generation 1 azide derivative **4** (400 MHz ^1^H NMR, 101 MHz ^13^C NMR in CDCl_3_).

By NMR it was possible to observe how the azide derivative signals changed from one generation to the other (Figure 2). In the case of **6,** we observed a new ^1^H NMR signal around 1.25 ppm (Figure 2A, signal c) corresponding to the methyl groups of the generation 2 dendrimer, as well as a new carbonyl carbon in the ^13^C NMR (Figure 2A, signal e).

**Scheme 2:**
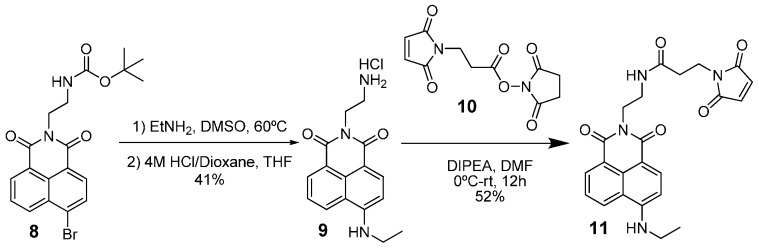
Synthesis of naphthalimide derivative **11**.

With the dendrimeric structure obtained, the next step was to modify the chromophore in order to couple the two fragments. Synthesis of the naphthalimide derivative is summarized in Scheme 2. After formation of 4-amino-1,8-naftalimide with ethylamine in DMSO and amine deprotection in HCl dioxane, we obtained compound **9** which presents the amino group needed for functionalization and subsequent coupling to the dendrimeric structure. Suitable functionalization was achieved using a maleimide ring, which shows orthogonal reactivity with the thiol group. This allows a new C–S bond to be established between the chromophore and the dendrimeric structure via a click reaction employing a 1,4-Michael addition on the double bond [33,34]. The maleimide ring was added to the naphthalimide by a reaction between the free amino group of the chromophore, **9**, and succinimidyl ester, **10** (Scheme 2).

**Scheme 3:**
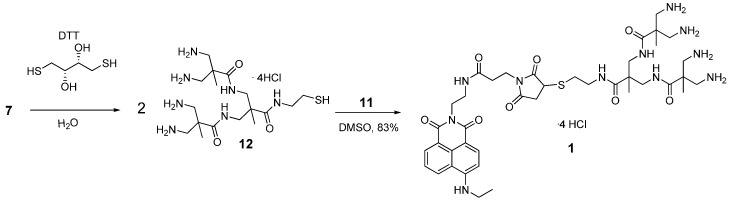
Synthesis of fluorescent dendron **1**.

The final step consists in the reduction of the disulfide bond of dendrimer **7** using dithiothreitol (DTT) to obtain two dendrons with a thiol group in the focal point, **12**. In the presence of the maleimide ring, the thiol group selectively attacks the double bond, resulting in the fluorescent dendron **1** (Scheme 3).

**Figure 3:**
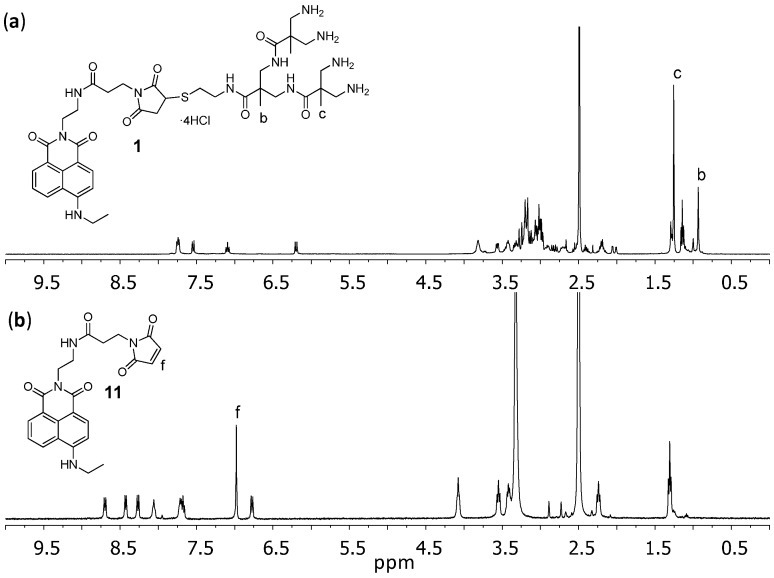
NMR comparison between: (**a**) fluorescent dendron **1** and (**b**) naphthalimide derivative **11** (400 MHz ^1^H NMR in DMSO-*d*_6_).

The structure of dendron **1** was elucidated by NMR and high-resolution mass spectrometry. The characteristic ^1^H RMN signal of the maleimide ring double bond disappeared in dendron **1**, confirming the addition of the thiol group (Figure 3). The signals corresponding to the dendrimeric structure can be observed in the spectrum, especially those belonging to the methyl groups of the two generations around 1.25 ppm (Figure 3A, signal c) and 1.00 ppm (Figure 3A, signal b), respectively.

**Scheme 4:**
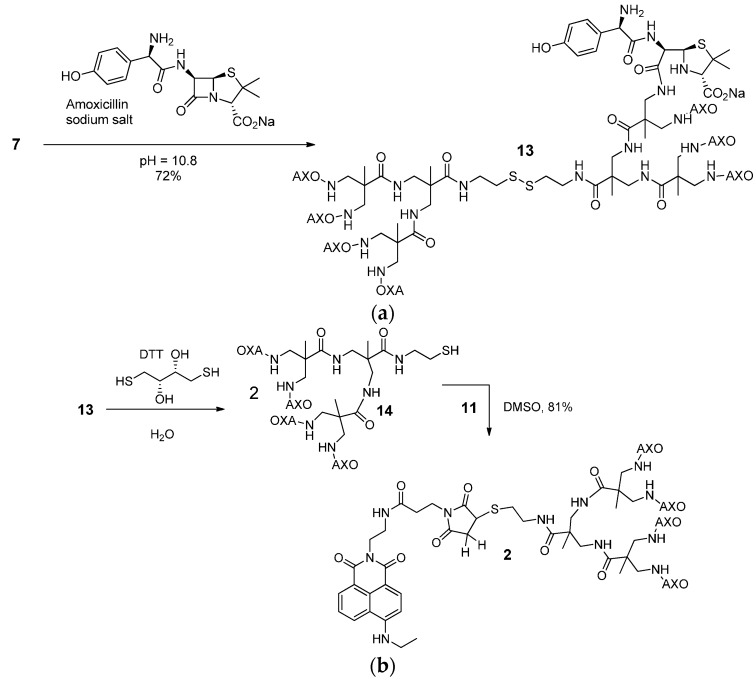
(**a**) Synthesis of dendrimeric antigen **13**; (**b**) synthesis of fluorescent dendron **2**.

From this fluorescent dendron, functionalization of the ammonium groups was tested using amoxicillin by inducing the attack of the amino groups to the betalactamic ring. However, the reaction did not proceed. An alternative method consisting of the functionalization of dendrimer **7** was first carried out. To a solution of **7** in water at pH = 10.8, amoxicillin in its sodium form was added in portions over 48 h. The resulting dendrimeric antigen **13**, was then purified by gel filtration chromatography, Sephadex G-10 in water (Scheme 4a).

Once the dendrimeric structures with the correct functionalization were obtained, the same protocol used to obtain **1** was applied but using **13** as the starting dendrimer. This generated a second fluorescent dendron, **2** (Scheme 4b).

**Figure 4:**
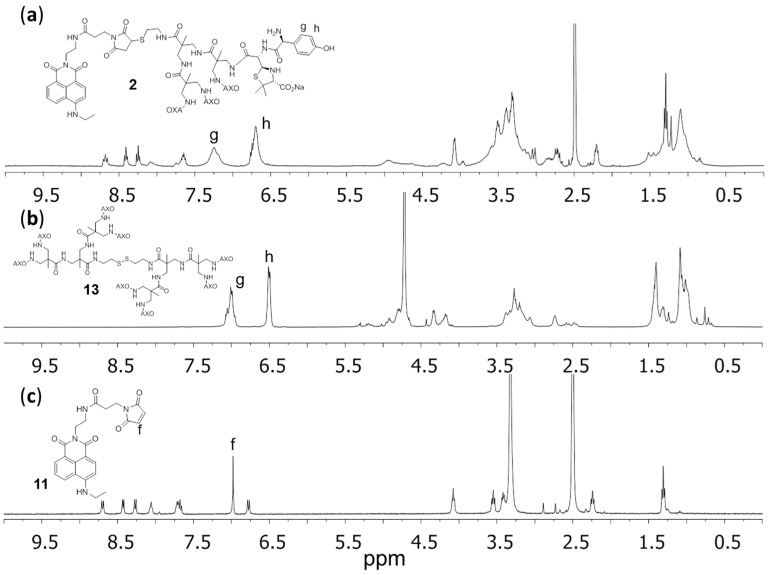
NMR comparison between: (**a**) fluorescent dendron **2** in DMSO-*d*_6_; (**b**) dendrimeric antigen **13** in D_2_O and (**c**) naphthalimide **11** in DMSO-*d*_6_ (400 MHz ^1^H NMR).

Disappearance of the double bond ^1^H NMR signal upon thiol group addition to the maleimide ring could again be detected. We also observed signals corresponding to the amoxicilloyl group, in particular those from the phenyl group around 7.25 ppm (Figure 4a, signal g) and 6.75 ppm (Figure 4a, signal h).

### 3.2. Fluorescent Properties Study

**Figure 5:**
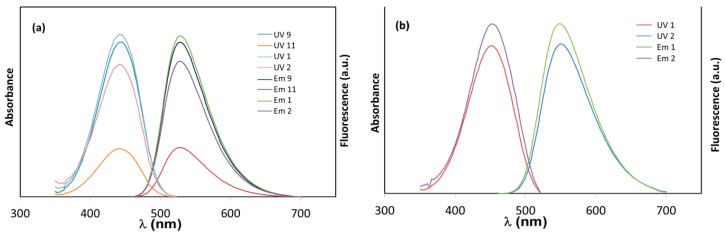
Absorption and fluorescence emission spectra of (**a**) **9**, **11**, **1**, and **2** in DMSO and (**b**) **1** and **2** in PBS (pH = 7.4).

Aside from their NMR and MS characterization, compounds **9**, **11**, **1**, and **2** have been characterized by their spectroscopic properties. Measurements of dendrons **1** and **2** were conducted in two solvents: PBS (pH = 7.4) and DMSO. These properties were compared with reference compounds (**9**, **11**) in DMSO. The absorption and fluorescence emission spectra in DMSO for all the compounds were registered (Figure 5). The absorption and fluorescence emission maximum are located around 442 and 528 nm, respectively, for all of the compounds studied. The Stokes shift for the electronic emission spectrum measured in this solvent was 3865 cm^−1^ in all cases. The values for fluorescent quantum yield (Φ_F_), using Rhodamine B as standard [35], and lifetime (τ), are presented in Table 1. The lower value of Φ_F_ for **11** is attributed to the deactivating presence of the maleimide ring, an effect observed in other fluorescent systems [36]. For the other three compounds the similar value of Φ_F_ indicates that the presence of the dendrimeric structure keeps the spectroscopic properties of the naphthalimide intact (Figures S32–35). Also, it is important to note that the phenyl ring present in the AXO group of dendron **2** does not overlap with the fluorescent emission of naphthalimide due to its higher energy.

**Figure 6:**
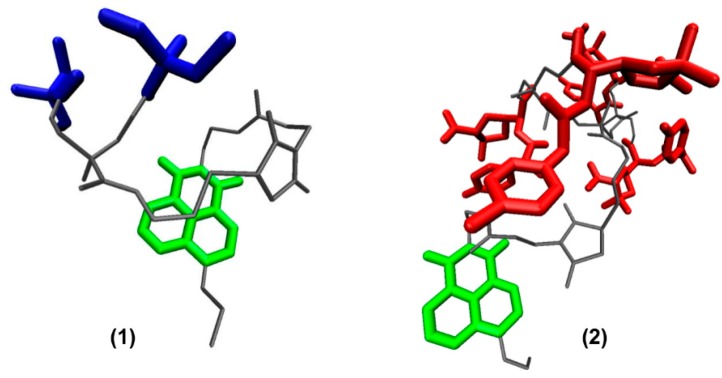
Selected snapshot of dendrons **1** and **2** after MD simulations. To simplify the figure, hydrogen atoms have been omitted, the spacer and repetitive BAPAD units are in grey, the naphthalimide moiety is in green, the amino terminal groups of **1** are in blue, and the amoxicilloyl groups of **2** are in red.

In PBS the absorption and fluorescence emission spectra for dendrons **1** and **2** were registered (Figure 6), showing maximums around 452 and 549 nm, respectively. The change of solvent causes a bathochromic shift from DMSO to PBS. The excited naphthalimide state is polar, resulting in lower fluorescence emission in polar protic solvents [37]. Thus, the value of Φ_F_, for both dendrons was lower in aqueous solution (Table 1). The Stokes shift was calculated at 3942 cm^−1^.

Naphthalimides are fairly nonpolar molecules that in aqueous media tend to interact between themselves, establishing dimers and other agglomerations. The absorption and fluorescence emission spectra of a series of known dilutions of compounds **1** and **2** were registered (Figures S30–35 in the Supplementary Materials). The absence of significant deviations of absorption and fluorescence emission *vs.* concentration from linearity indicates that both spectra derive from a unique species with the same chemical surroundings within the concentration range studied, corresponding to the range typically used for this type of study.

### 3.3. Theoretical Calculations of Compounds **1** and **2**

In order to obtain information about the size and shape of these compounds in a biological medium, molecular models were created for dendrons **1** to **2** and simulated in water as explicit solvent using molecular dynamics.

These dendrons were built with three different residues: the core (COR), which includes the naphthalimide fluorophore and the spacer toward the BAPAD unit, the branched repeating fragment (REP), and the terminal ends (TAM) and (AXO) for the amino and amoxicilloyl dendrons, respectively (Figure S36 in the Supplementary Materials).

Relaxation of these molecules was determined from the autocorrelation function of the squared radius of gyration (Figure S37 in the Supplementary Materials) and the equilibrated structures (Figure 6) were analyzed. Dendrimer size was quantified considering the radius-of-gyration (*R*_g_) and the radius obtained from the variations of the accessible surface area (*R*_SASA_) (Figures S38 and S39 in the Supplementary Materials). Shapes were obtained from the ratio between the three principal moments of inertia (*I_x_*, *I_y_*, and *I_z_*) and their asphericities (see initial properties in Table S1). The obtained values are shown in Table 2.

As can be inferred from their *R*_SASA_, which can represent the effective macromolecule size, the introduction of the fluorophore with the link to dendron **1**, results in a size similar to the corresponding G2-amino-dendrimer [4]. For compound **2**, introduction of the amoxicilloyl moiety increased it to a size similar to that of the G3-amino-dendrimer.

Compared to the corresponding amino BAPAD-dendrimers [4], the eccentricity and asphericity of both dendrons suggest that they have a more spherical shape, probably due to the presence of the uncharged fluorophore.

**Figure 7:**
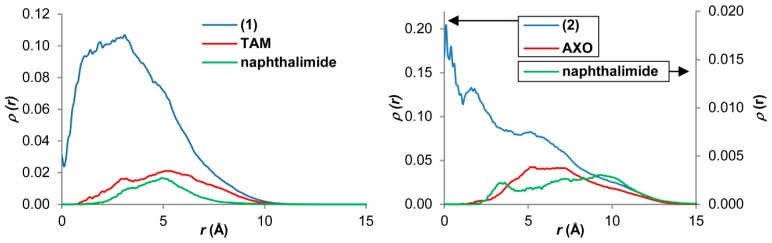
Radial distribution function of dendrons **1** and **2**, their corresponding terminal groups and the naphthalimide moiety using dendrimer center-of-mass as a reference. The unit value for *ρ(r)* is expressed in atoms/Å^3^.

The distribution of different residues within molecules can be described by their radial density profiles (Figure 7 and Figure S40 in the Supplementary Materials). For dendrons **1** and **2**, the probability of finding the terminal monomers (TAM or AXO) always tends toward the periphery of the molecules, making these residues more exposed to the solvent. The naphthalimide moiety is also positioned toward the periphery in a similar way (Figure 6).

### 3.4. Biomedical Applications of Compounds **1** and **2**

Antigen-presenting cells, such as dendritic cells (DCs), are the first to make contact, process, and present antigens to the immune system. When an antigen is recognized as a potential danger, DC undergo a maturation process where both the antigen and co-estimulatory molecules are presented on the mature cell surface. The interaction between mature DCs and other immune system cells (such as lymphocytes) induces their activation [38] and initiates an immune response. Where this process is activated abnormally, it can result in allergic responses. Immature DCs (iDCs) can be obtained from monocytes isolated from peripheral blood of tolerant and allergic subjects following established protocols [39]. This allows the allergic responses of iDCs from allergic and non-allergic (tolerant) patients to be studied *in vitro*. To understand the ability of BAPAD-dendrimers to act as biological scaffolds. We tested their ability to be internalized by antigen-presenting cells. To that end, we next looked at interactions between iDCS and compounds **1** and **2**.

**Figure 8:**
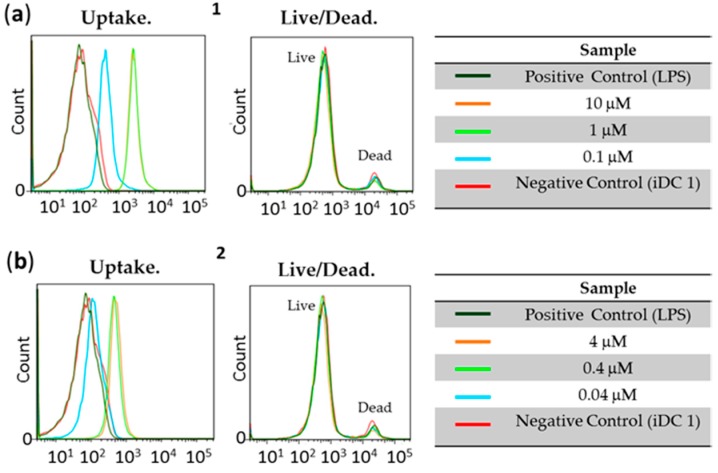
Flow cytometry histograms: (**a**) Uptake and Live/Dead ratio for **1**; (**b**) uptake and Live/Dead ratio for **2**.

To analyze the capacity of the compounds to be taken up by DCs, iDCs from tolerant patients were used. After incubation, cells were studied with a flow cytometer to determine whether or not the cells had been able to take up the compounds and if that process had provoked cell death. The flow cytometry data are presented in the form of histograms for the two compounds (Figure 8). The *y* axis represents the number of cells detected and the *x* axis the fluorescence emission intensity. iDCs were incubated at 0.1, 1.0, and 10 μM for **1** and 0.04, 0.4, and 4 μM for **2**. Incubation without stimulation was used as a negative uptake control and incubation with lipopolysaccharides (LPS) as a positive control.

**Figure 9:**
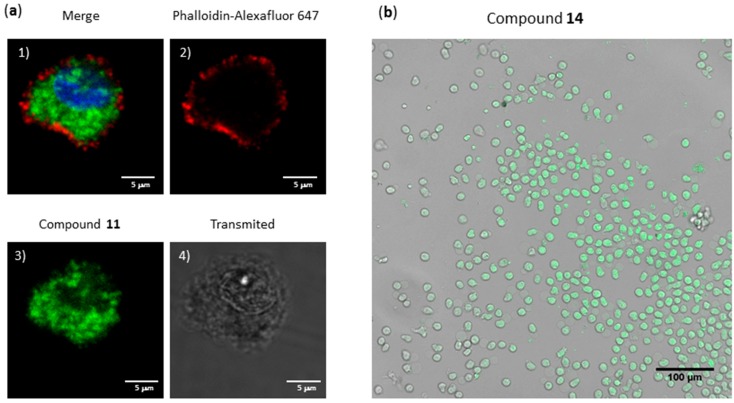
Confocal microscope images of DCs fixed after incubation with compounds **1** and **2** then stained with DNA- (Hoescht; blue) and actin- (Phallodin; Red) specific stains to complement naphthalimide fluorescence (green): (**a**) Detail of a DC incubated with **1**: (1) Combination of all measured channels including; (2) Phalloidin-Alexafluor 647 cortical stain; (3) Naphthalimide fluorescence (4) Transmitted light (non-confocal); (**b**) DC population incubated with **2**, combined naphthalimide fluorescence emission, and transmitted light channels.

The data from the flow cytometry experiments after an incubation of 48 h indicate that both compounds can enter inside the cells and that even for high levels of the two compounds cell viability is barely affected. The uptake experiments performed showed that the fluorescence emission intensity increases with concentration. For compound 1 the major increase occurs from 0.01 to 0.1 μM and it is practically unchanged from 1.0 to 10 μM. The same behavior is observed for compound **2**. By visualizing DCs that had been fixed after incubation using a confocal microscope, it was possible to follow where the compounds had accumulated in the cell (Figure 9). The characteristic fluorescence of naphthalimide could readily be detected in the cytoplasm of the cells.

**Figure 10:**
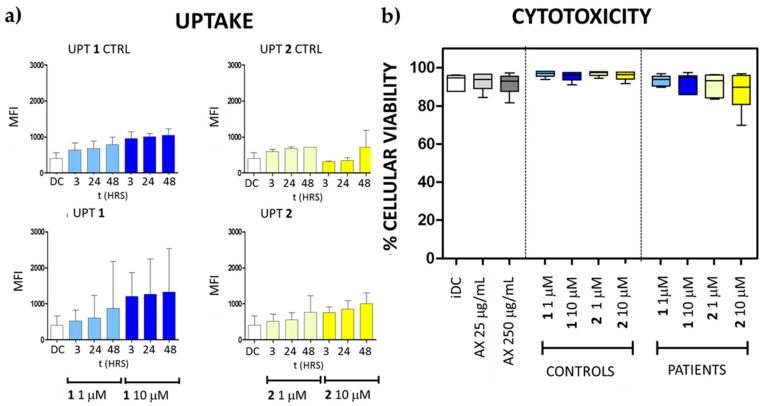
Flow cytometry results for incubation with DCs from allergic patients: (**a**) Uptake; (**b**) cytotoxicity.

**Figure 11:**
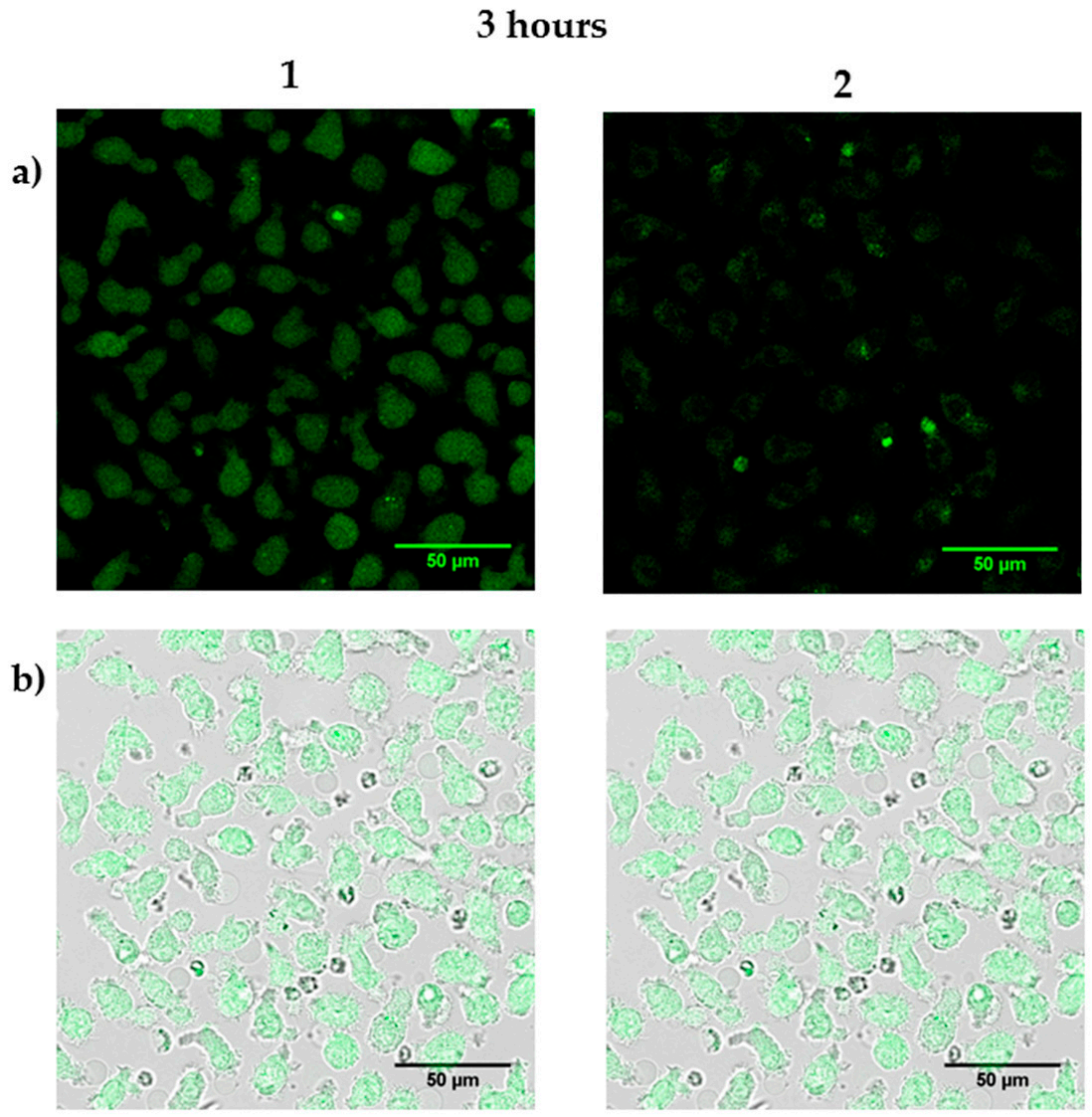
Confocal microscope images of DCs incubated with **1** and **2** (1 mM): (**a**) Naphthalimide fluorescence; (**b**) merged fluorescence and transmitted light channels.

After confirming the cellular uptake by DCs of both compounds and the absence of high mortality among cell populations, the next step was to analyze the effect of incubating them with cells from allergic patients at different time-points. As before, the uptake of the compounds and their cytotoxicity was obtained by flow cytometry (Figure 10). Uptake experiments, in terms of median fluorescence intensity (MFI) at different incubation times and at the two different concentrations (1 and 10 μM) that previously showed higher internalization rates, have been carried out. iDCs without the addition of either compound were used as the fluorescence-negative control. Confocal microscope images of the different populations were also captured (Figure 11).

Results from the uptake experiments were similar in allergic patients and tolerant subjects; however, in the former higher values of MFI were observed at higher concentrations. This fluorescence increases not only with the concentration, but with longer incubation times. Regarding cytotoxicity, the high cellular viability values obtained are evident, always higher than 80% after 48 h of incubation, even with the higher concentrations. All these results showed the biocompatibility of these compounds and their potential for *in vitro* studies in tolerant subjects and allergic patients, allowing their visualization inside the cells.

## 4. Conclusions

We have shown here that the combination of the properties of a new model of dendrons (BisAminoalkylPolyAmide Dendrimers or BAPAD) with a fluorescent label represents a powerful tool for biomedical studies. We have validated our synthetic approach and fully characterized the resulting structures. In addition, we have demonstrated using *in vitro* experiments with human dendritic cells that these structures are not toxic, internalize efficiently inside dendritic cells, and localize preferentially in the cytoplasm. Our data show that BAPAD-based structures are robust tools for biomedical studies and show promise as synthetic biocompatible scaffolds with a wide range of potential biomedical applications.

## Figures and Tables

**Table 1:** Spectroscopic properties of **9**, **11**, **1**, and **2** in DMSO and of **1** and **2** in PBS (pH = 7.4).

Compound	Φ_F_	τ	ε
**9**	0.62 ^1^	10 ^1^	–
**11**	0.25 ^1^	4 ^1^	–
**1**	0.62 ^1^ 0.18 ^2^	10 ^1^ 4 ^2^	7738 ^2^
**2**	0.57 ^1^ 0.12 ^2^	10 ^1^ 4 ^2^	13,602 ^2^

^1^ DMSO; ^2^ PBS (pH = 7.4).

**Table 2:** Aspect ratios, asphericities, Radius-of-gyration (*R*g) and Radius of the Solvent Accessible Surface Area (*R*_SASA_) of the dendrons. ^a^

Dendron	*Ix/Iy*	*Ix/Iz*	δ	*R*g (Å)	*R*_SASA_ (Å)
**1**	1.18 ± 0.04	2.68 ± 0.35	0.064 ± 0.014	5.94 ± 0.18	7.47
**2**	1.30 ± 0.07	1.64 ± 0.17	0.021 ± 0.007	8.56 ± 0.41	11.49

^a^ Values averaged over the last 1 ns simulation run. Each snapshot was taken after 1 ps.

## Supplementary Materials

The following are available online at www.mdpi.com/2073-4360/8/4/111/s1. Figure S1: ^1^H NMR spectrum (400 MHz) of 1 in D2O; Figure S2: ^13^C NMR (101 MHz) of **1** in D_2_O with CDCl_3_ as reference; Figure S3: ^1^H NMR spectrum (600 MHz) of **2** in DMSO-*d*_6_; Figure S4: ^13^C NMR spectrum (151 MHz) of **2** in DMSO-*d*_6_; Figure S5: ^1^H NMR spectrum (400 MHz) of **3** in CDCl_3_; Figure S6: ^13^C NMR (101 MHz) of **3** in CDCl_3_; Figure S7: ^1^H NMR spectrum (400 MHz) of **4** in CDCl_3_; Figure S8: ^13^C NMR (101 MHz) of **4** in CDCl_3_; Figure S9: ^1^H NMR spectrum (400 MHz) of **5** in D_2_O.; Figure S10: ^13^C NMR spectrum (101 MHz) of **5** in DMSO-*d*_6_; Figure S11: ^1^H NMR spectrum (400 MHz) of **6** in CDCl_3_; Figure S12: ^13^C NMR (101 MHz) of **6** in CDCl_3_; Figure S13: ^1^H NMR spectrum (400 MHz) of **7** in D_2_O; Figure S14: ^13^C NMR (101 MHz) of **7** in D_2_O with CDCl_3_ as reference; Figure S15: ^1^H NMR spectrum (400 MHz) of **9** in CDCl_3_; Figure S16: ^13^C NMR (101 MHz) of **9** in CDCl_3_; Figure S17: ^1^H NMR spectrum (400 MHz) of **9** in DMSO-*d*_6_; Figure S18: ^13^C NMR (101 MHz) of **11** in DMSO-*d*_6_; Figure S19: ^1^H NMR spectrum (400 MHz) of **13** in D_2_O; Figure S20: ^13^C NMR (101 MHz) of **13** in D_2_O; Figure S21: MALDI-TOF-MS of compound **1**; Figure S22: MALDI-TOF-MS of compound **2**; Figure S23: HRMS ESI of compound **4**; Figure S24: HRMS ESI of compound **5**; Figure S25: ESI-MS of compound **6**; Figure S26: MALDI-TOF-MS of compound **7**; Figure S27: ESI-MS of compound **9**; Figure S28: ESI-MS of compound **11**; Figure S29: MALDI-TOF-MS of compound **13**; Figure S30: Absorption and fluorescence emission spectra of **1** at different concentrations in PBS; Figure S31: Absorption maximum depending on the concentration of **1** in PBS (*R*^2^ = 0.9946); Figure S32: Fluorescence emission maximum depending on the concentration of **1** in PBS (*R*^2^ = 0.996); Figure S33: Absorption and fluorescence emission spectra of **2** at different concentrations in PBS; Figure S34: Absorption maximum depending on the concentration of **2** in PBS (*R*^2^ = 0.9985); Figure S35: Fluorescence emission maximum depending on the concentration of **2** in PBS (*R*^2^ = 0.9948); Figure S36: Residue selection for dendrons. Cap atoms are shown in red; Figure S37: Correlation functions of the squared radius of gyration for dendrons **1** and **2**; Figure S38: Time evolution of the Radius of Gyration against the last nanosecond for dendrons **1** and **2**; Figure S39: Square root of Solvent Accessible Surface Areas (SASA) as a function of probe radius *p* for dendrons **1** and **2**. A theoretical slope (4π)^1/2^ = 3.54 was used; Figure S40: Radial distribution function of dendrons **1** and **2**, with its monomers using dendrimer center of mass as reference. The unit value for *ρ (r)* is expressed in atoms/Å^3^; Table S1: Initial properties of dendrons and simulation details. *N*_den_, *N*_Cl_^−^, *N*_Na_^+^, *N*_water_ and *N*_total_ are, respectively, the number of dendron atoms, chloride ions, sodium ions, atoms in solvent molecules, and the total number of atoms. *V* is the initial octahedral box volume.
