# Supplementary material for: Fluorescent BAPAD Dendrimeric Antigens Are Efficiently Internalized by Human Dendritic Cells
Source: Polymers (Basel). 2016 Mar 26;8(4):111. doi: 10.3390/polym8040111 (PMC6432222; doi:10.3390/polym8040111)
Supplement: Supplementary file 1 [file polymers-08-00111-s001.pdf]

Supplementary Materials: Fluorescent BAPAD Dendrimeric Antigens Are Efficiently Internalized by Human Dendritic Cells

Pablo Mesa-Antúnez, Daniel Collado, Yolanda Vida, Francisco Najera Tahia Fernandez, Maria Jose Torres and Ezequiel Perez-Inestrosa

Content

^{1}H NMR, ^{13}C NMR Spectra	S1
MALDI-MS, HRMS ESI and ESI-MS of Compounds **1**, **2**, **4**, **5**, **6**, **7**, **9**, **11** and **13**	S11
Absorption and Fluorescence Emission Spectra of **1**, **2** at Different Concentration	S19
Molecular Dynamics Simulations Details	S21

^{1}H NMR, ^{13}C NMR Spectra

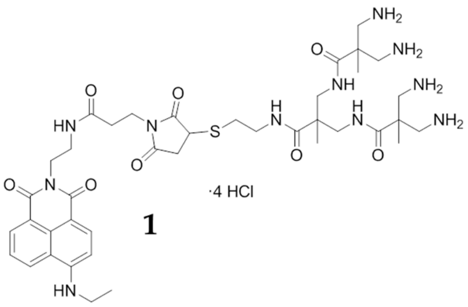

1

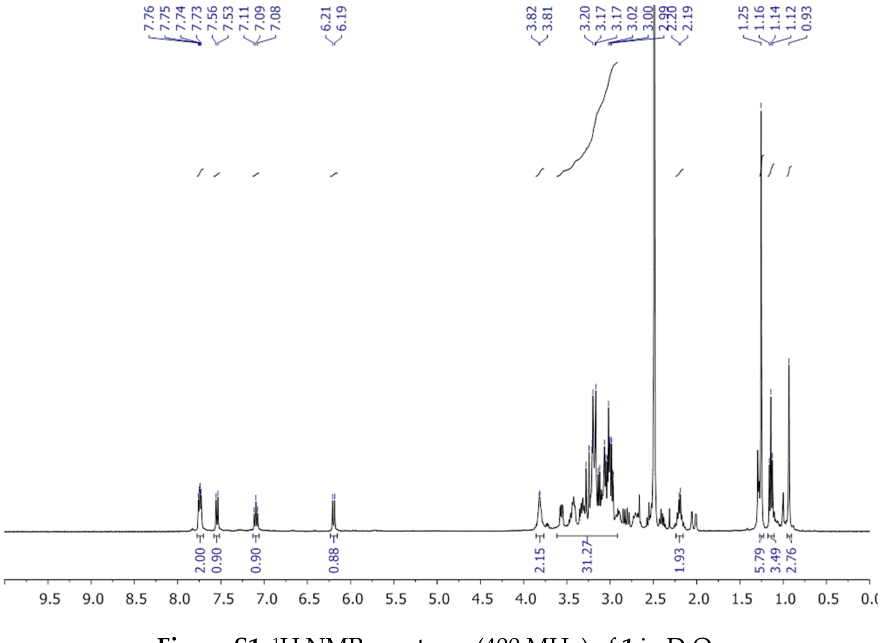

Figure S1. ^{1}H NMR spectrum (400 MHz) of **1** in D_2O.

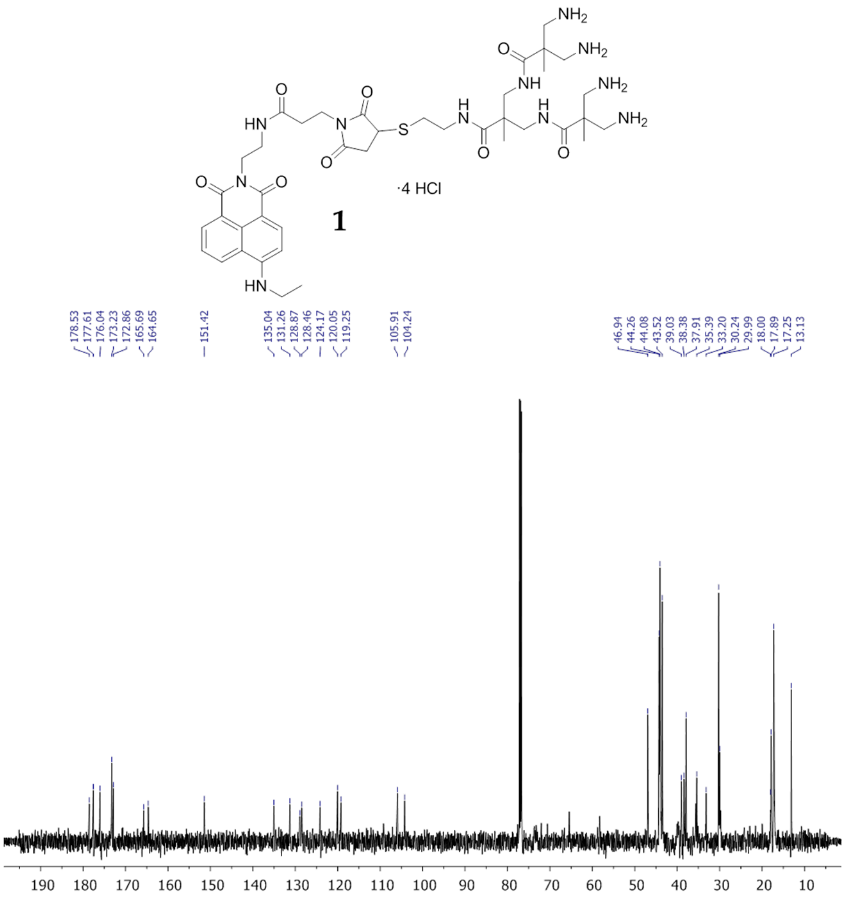

Figure S2. ^{13}C NMR (101 MHz) of **1** in D_2O with $CDCl_3$ as reference.

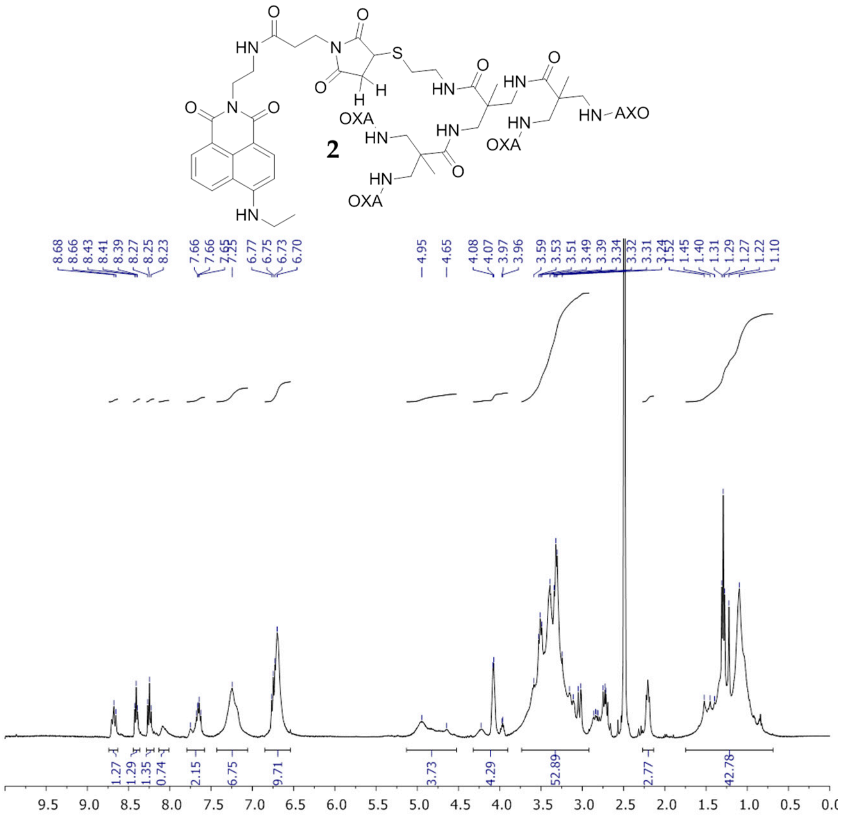

Figure S3. ^{1}H NMR spectrum (600 MHz) of **2** in DMSO-d_6.

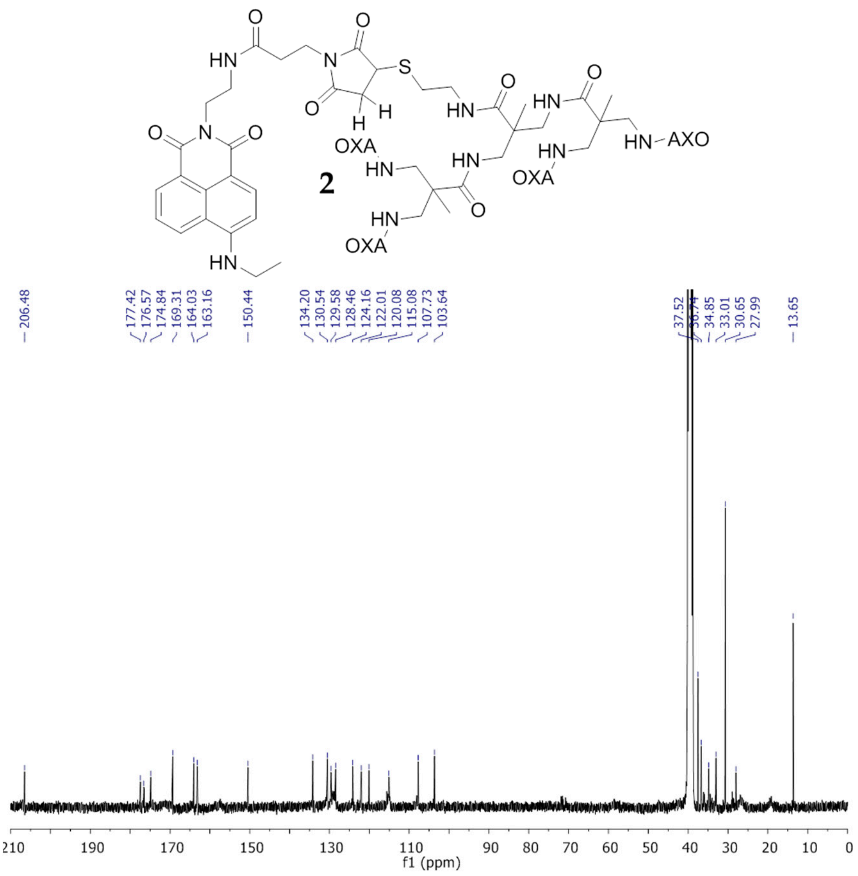

Figure S4. ^{13}C NMR spectrum (151 MHz) of **2** in DMSO-d_6.

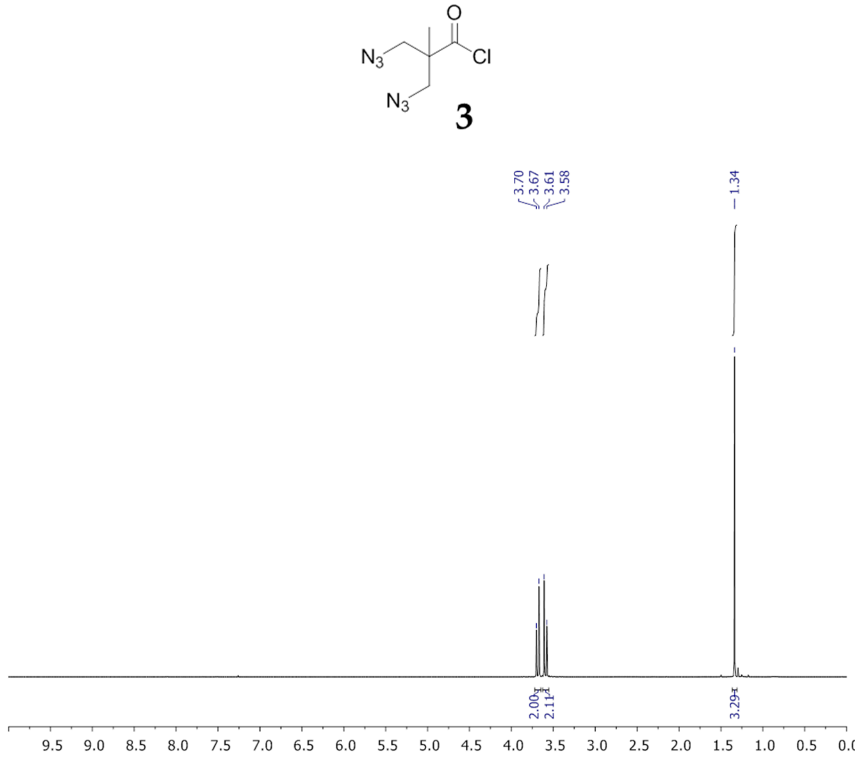

Figure S5. ^{1}H NMR spectrum (400 MHz) of **3** in $CDCl_3$.

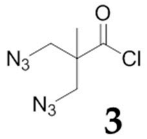

3

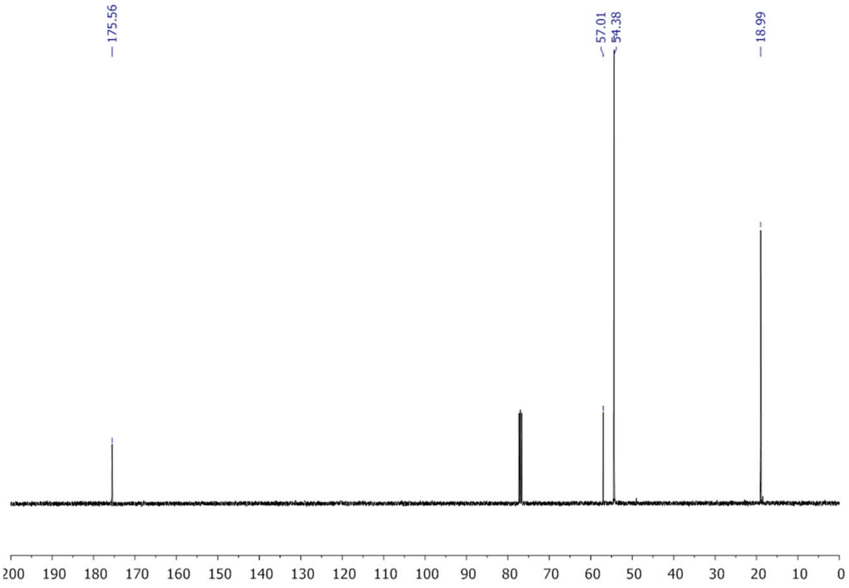

Figure S6. ^{13}C NMR (101 MHz) of **3** in $CDCl_3$.

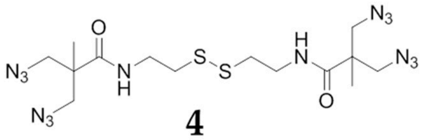

4

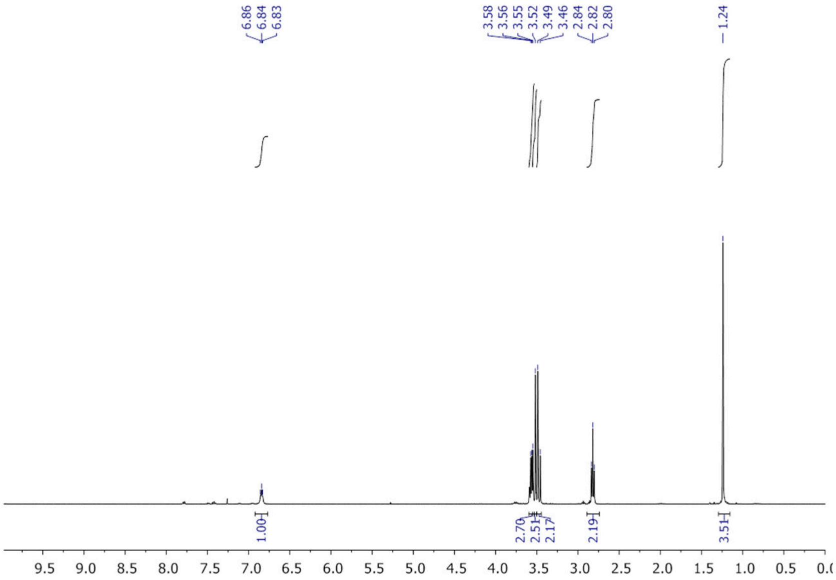

Figure S7. ^{1}H NMR spectrum (400 MHz) of **4** in $CDCl_3$.

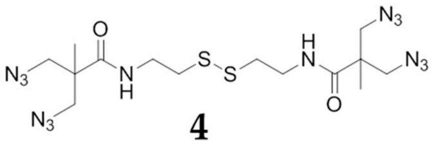

4

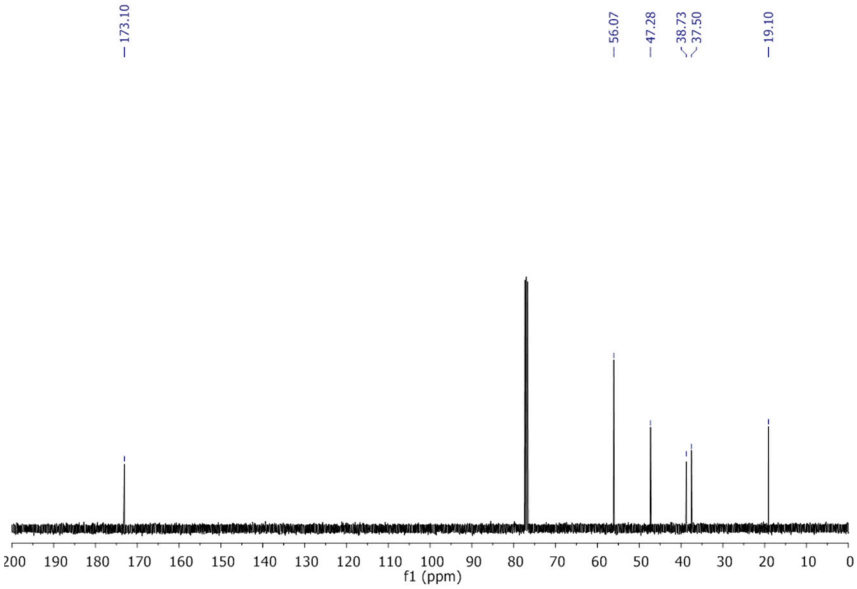

Figure S8. ^{13}C NMR (101 MHz) of **4** in $CDCl_3$.

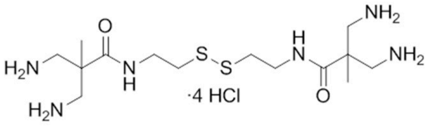

5

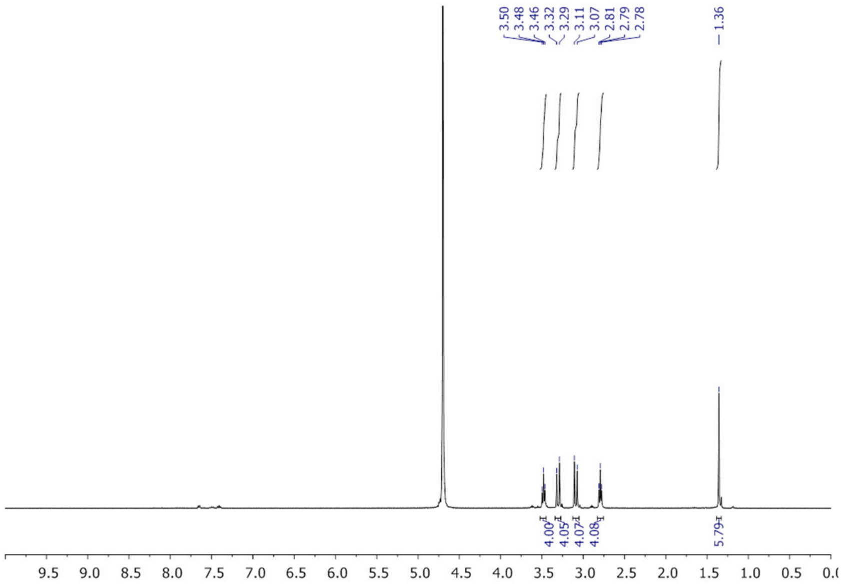

Figure S9. ^{1}H NMR spectrum (400 MHz) of **5** in D_2O.

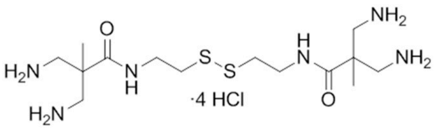

5

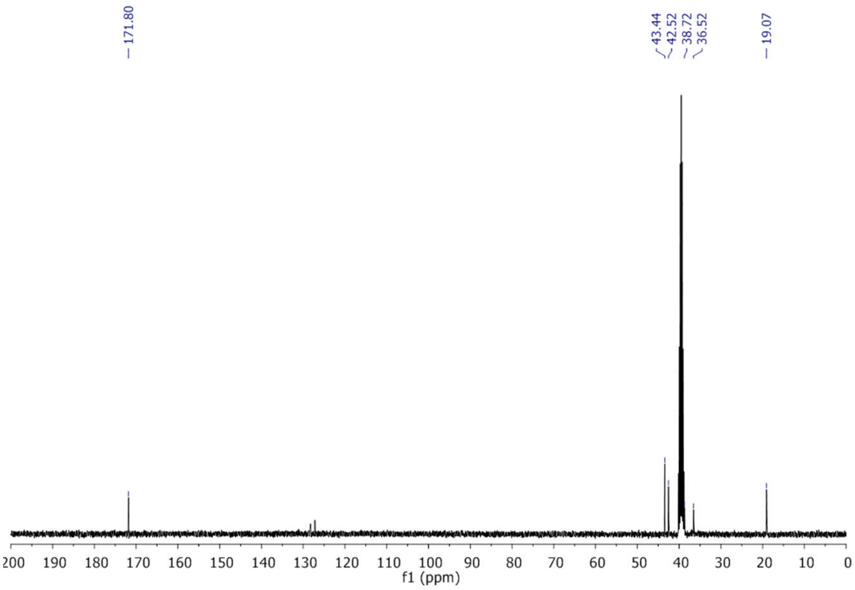

Figure S10. ^{13}C NMR spectrum (101 MHz) of **5** in DMSO-d_6.

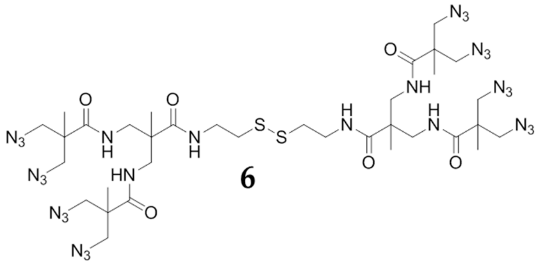

6

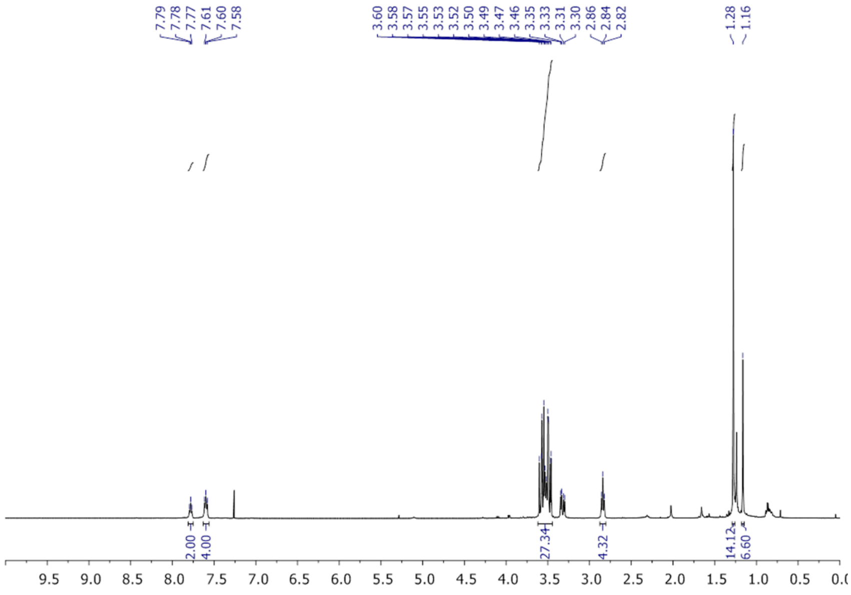

Figure S11. ^{1}H NMR spectrum (400 MHz) of **6** in $CDCl_3$.

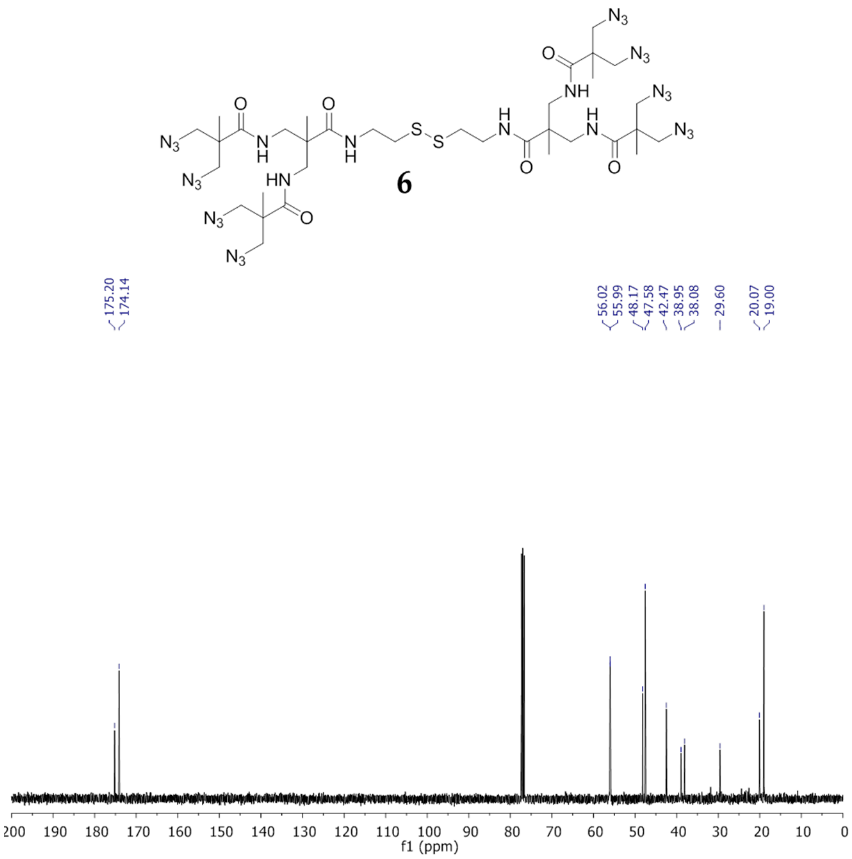

Figure S12. ^{13}C NMR (101 MHz) of **6** in $CDCl_3$.

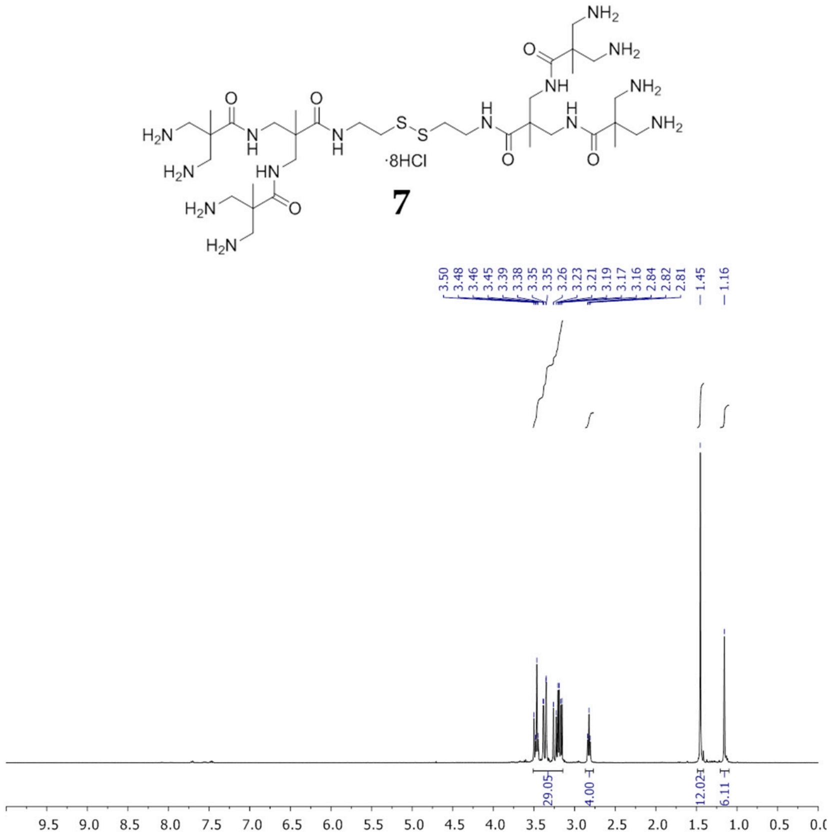

Figure S13. ^1H NMR spectrum (400 MHz) of **7** in D_2O.

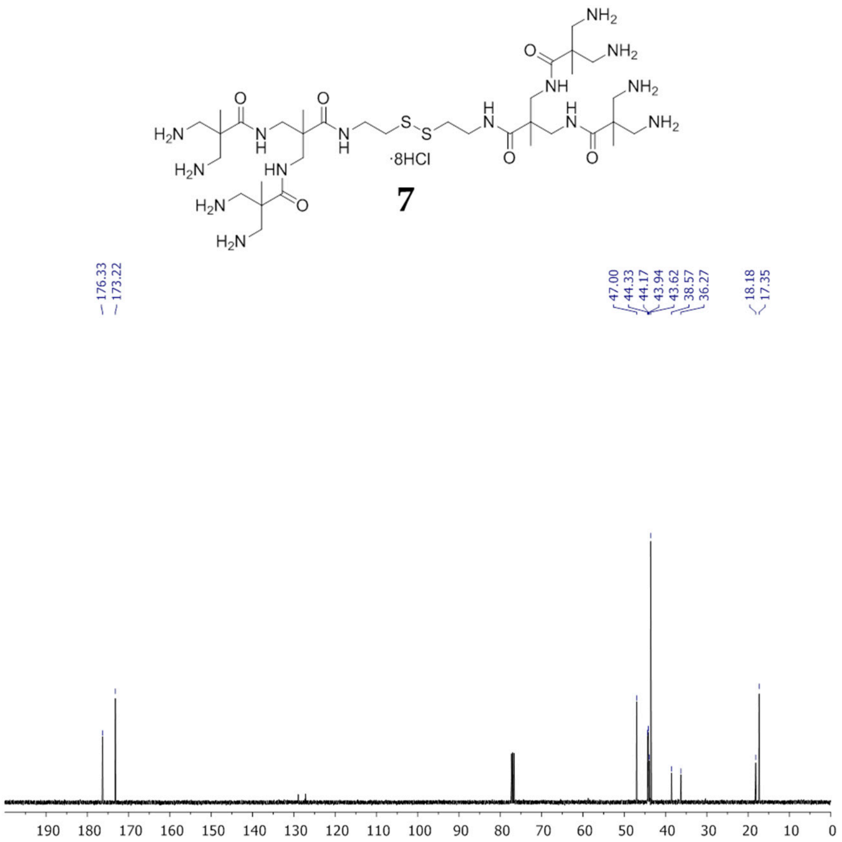

Figure S14. ^{13}C NMR (101 MHz) of **7** in D_2O with $CDCl_3$ as reference.

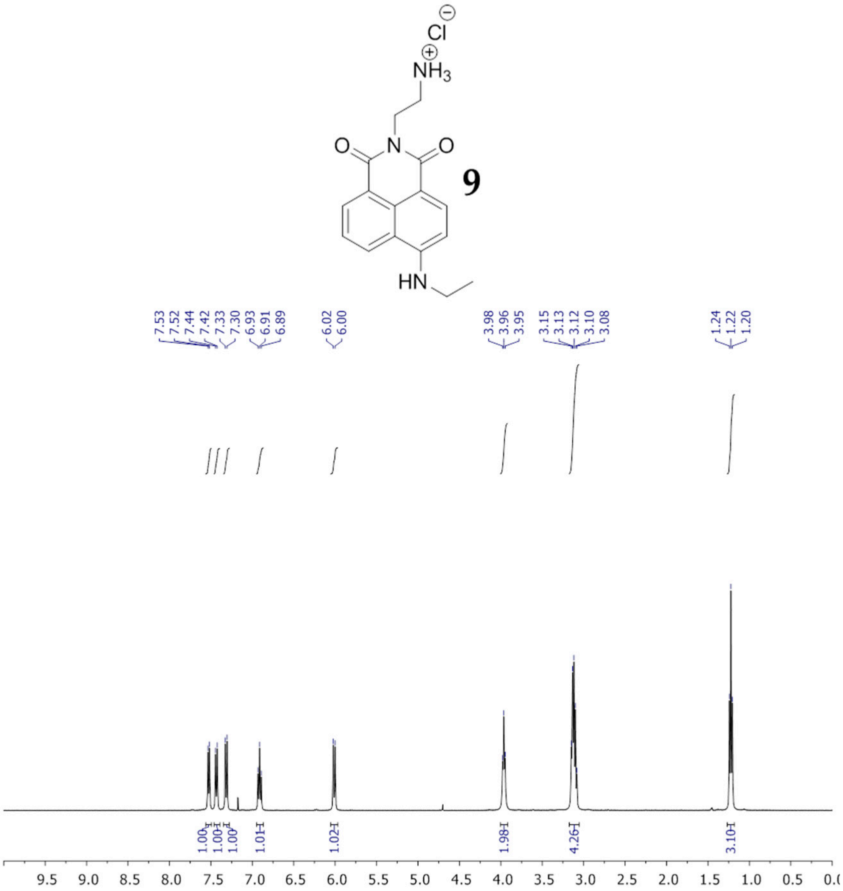

Figure S15. ^{1}H NMR spectrum (400 MHz) of **9** in $CDCl_3$.

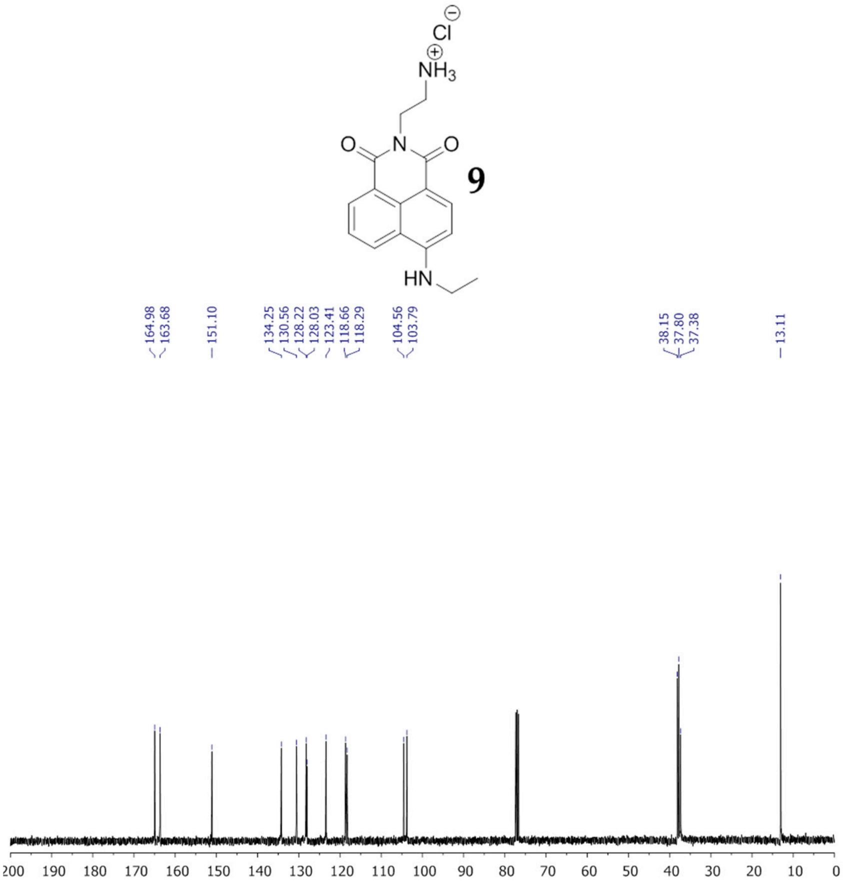

Figure S16. ^{13}C NMR (101 MHz) of **9** in $CDCl_3$.

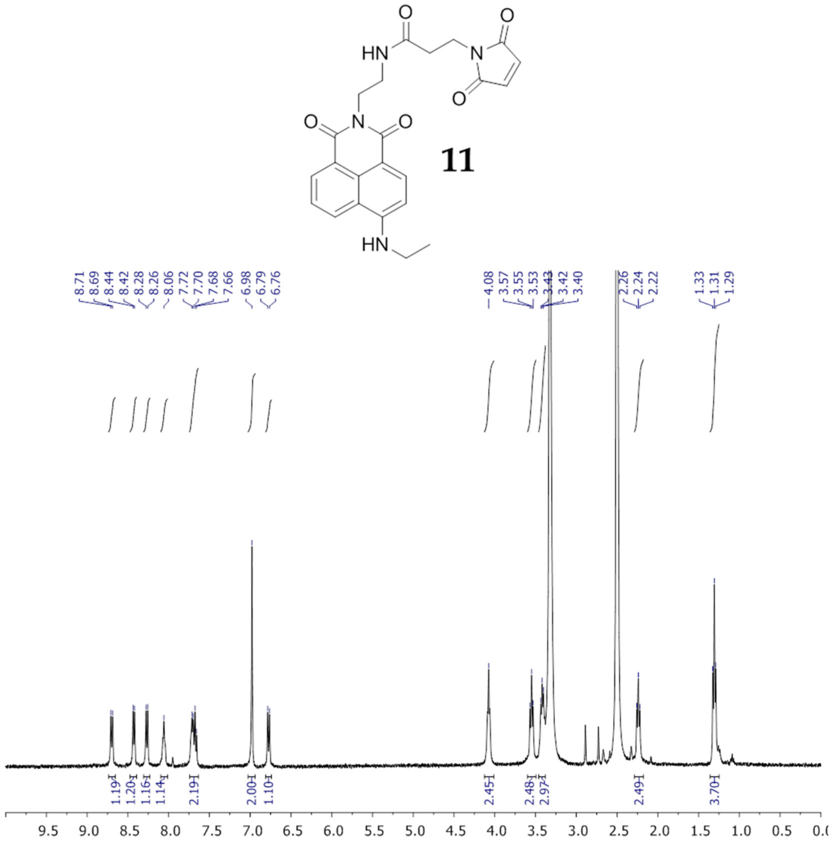

Figure S17. ^{1}H NMR spectrum (400 MHz) of **9** in DMSO-d_6.

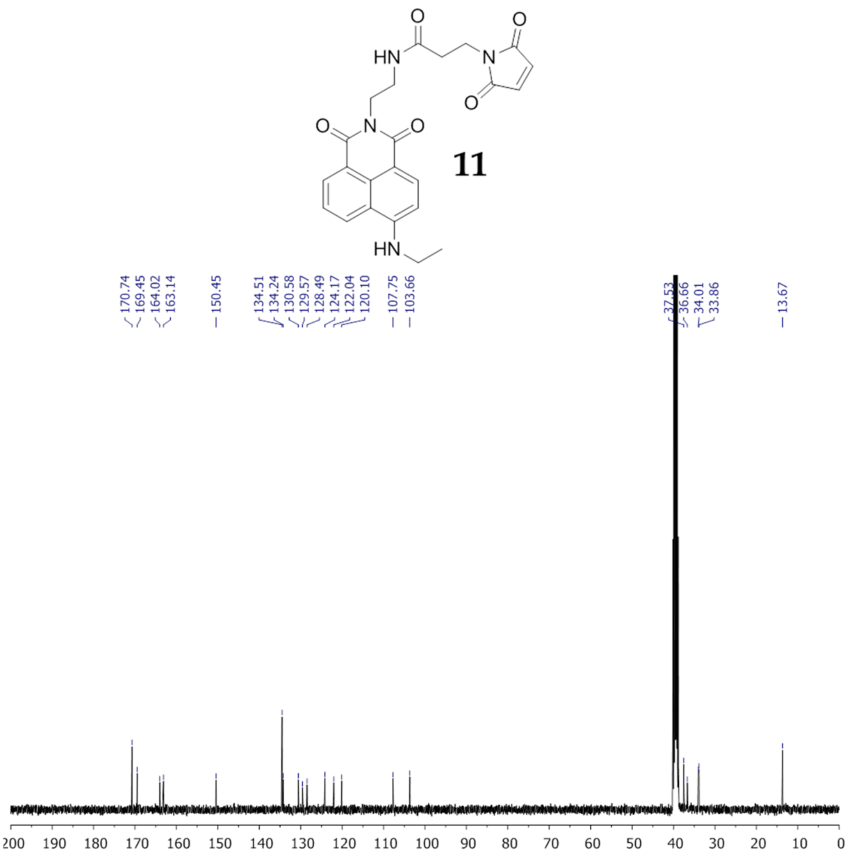

Figure S18. ^{13}C NMR (101 MHz) of **11** in DMSO-d_6.

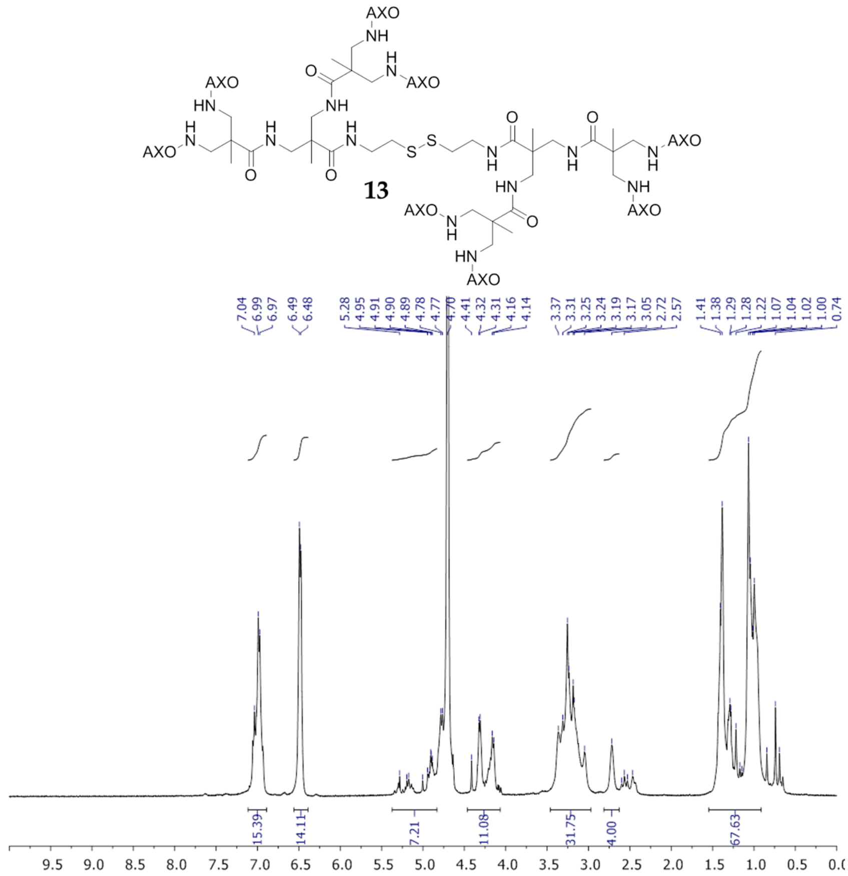

Figure S19. ^{1}H NMR spectrum (400 MHz) of **13** in D_2O.

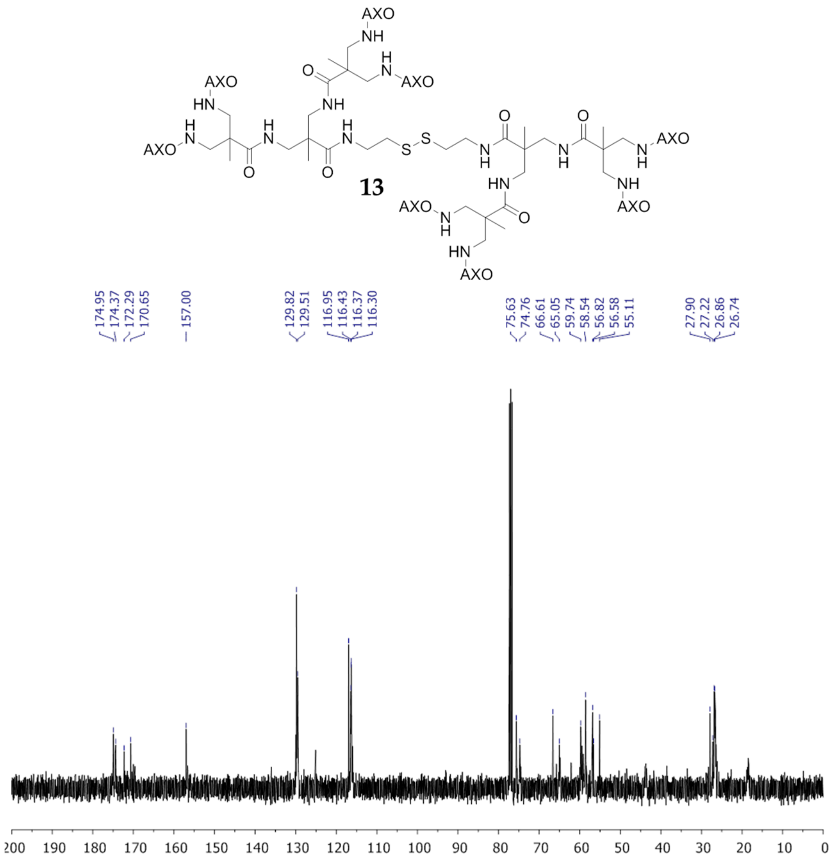

Figure S20. ^{13}C NMR (101 MHz) of **13** in D_2O.

MALDI-TOF-MS and ESI-MS of Compounds 1, 2, 4, 5, 6, 7, 9, 11 and 13

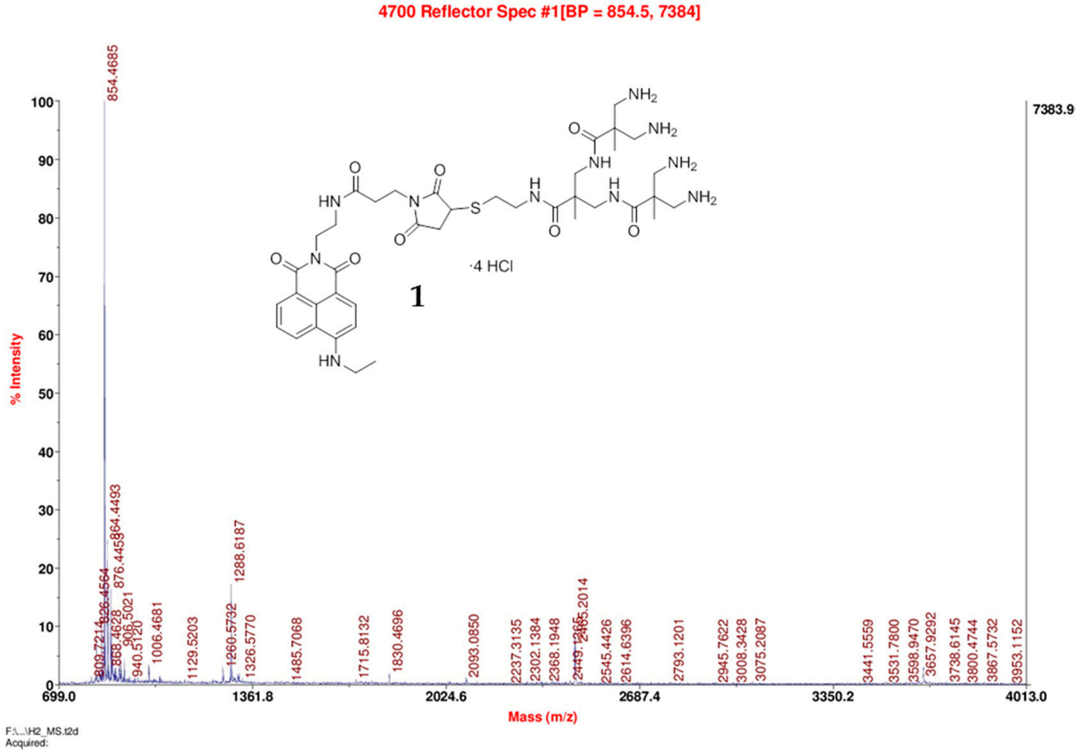

Figure S21. MALDI-TOF-MS of compound **1**.

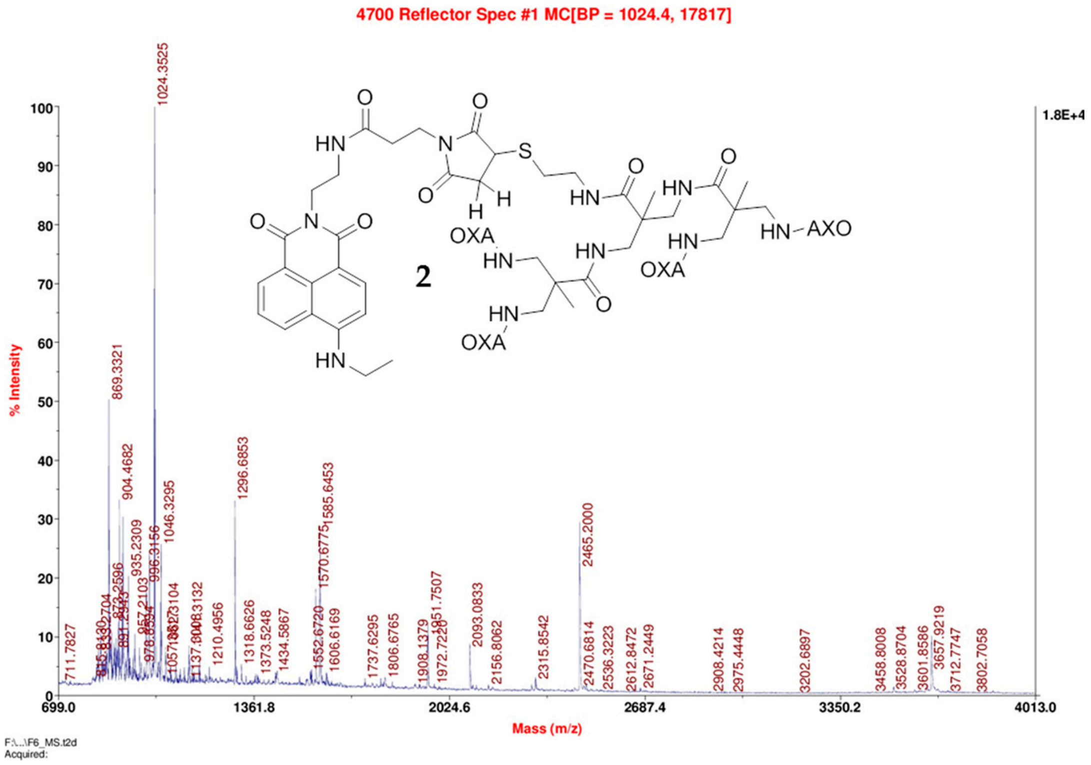

Figure S22. MALDI-TOF-MS of compound **2**.

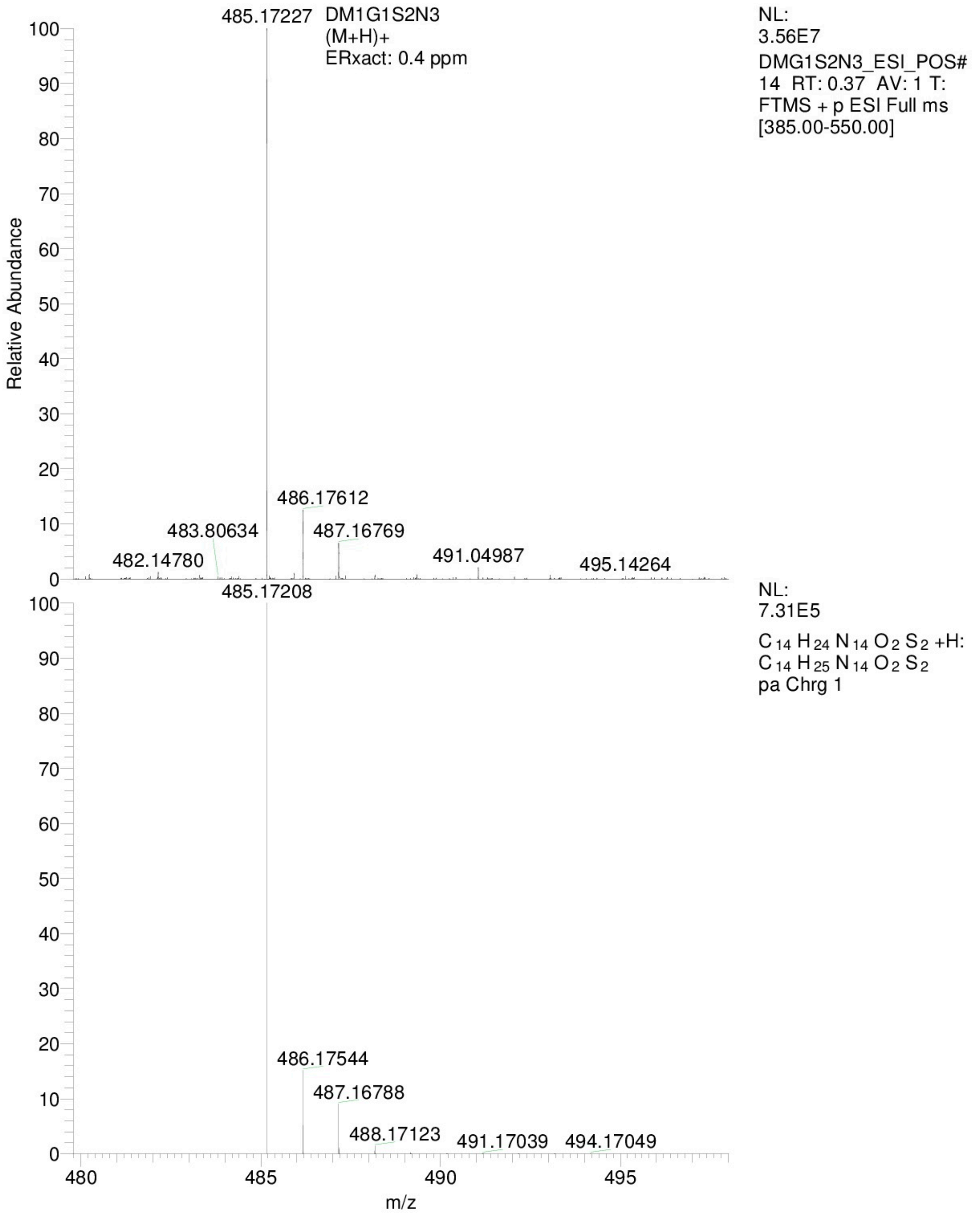

Figure S23. HRMS of compound **4**.

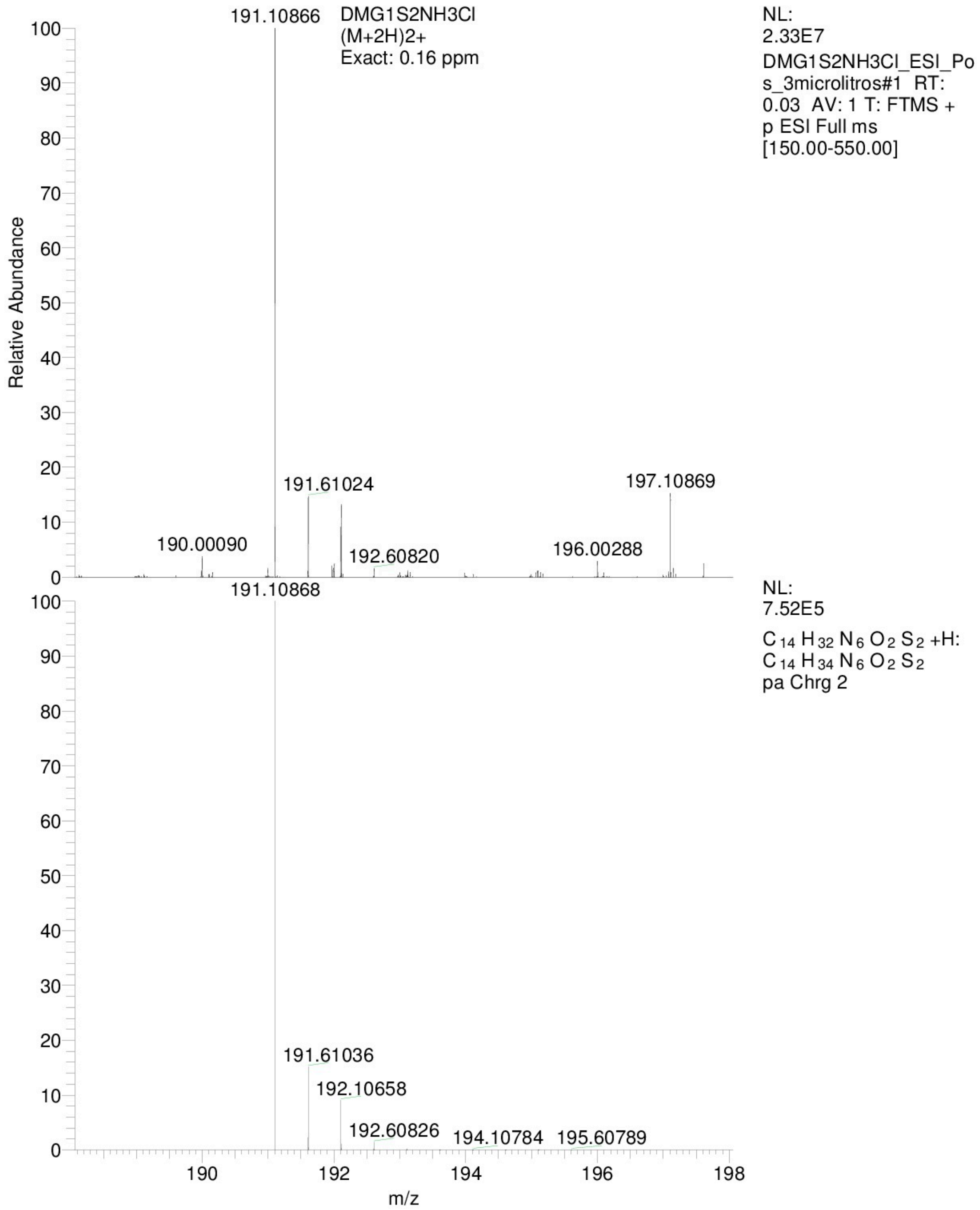

Figure S24. HRMS of compound **5**.

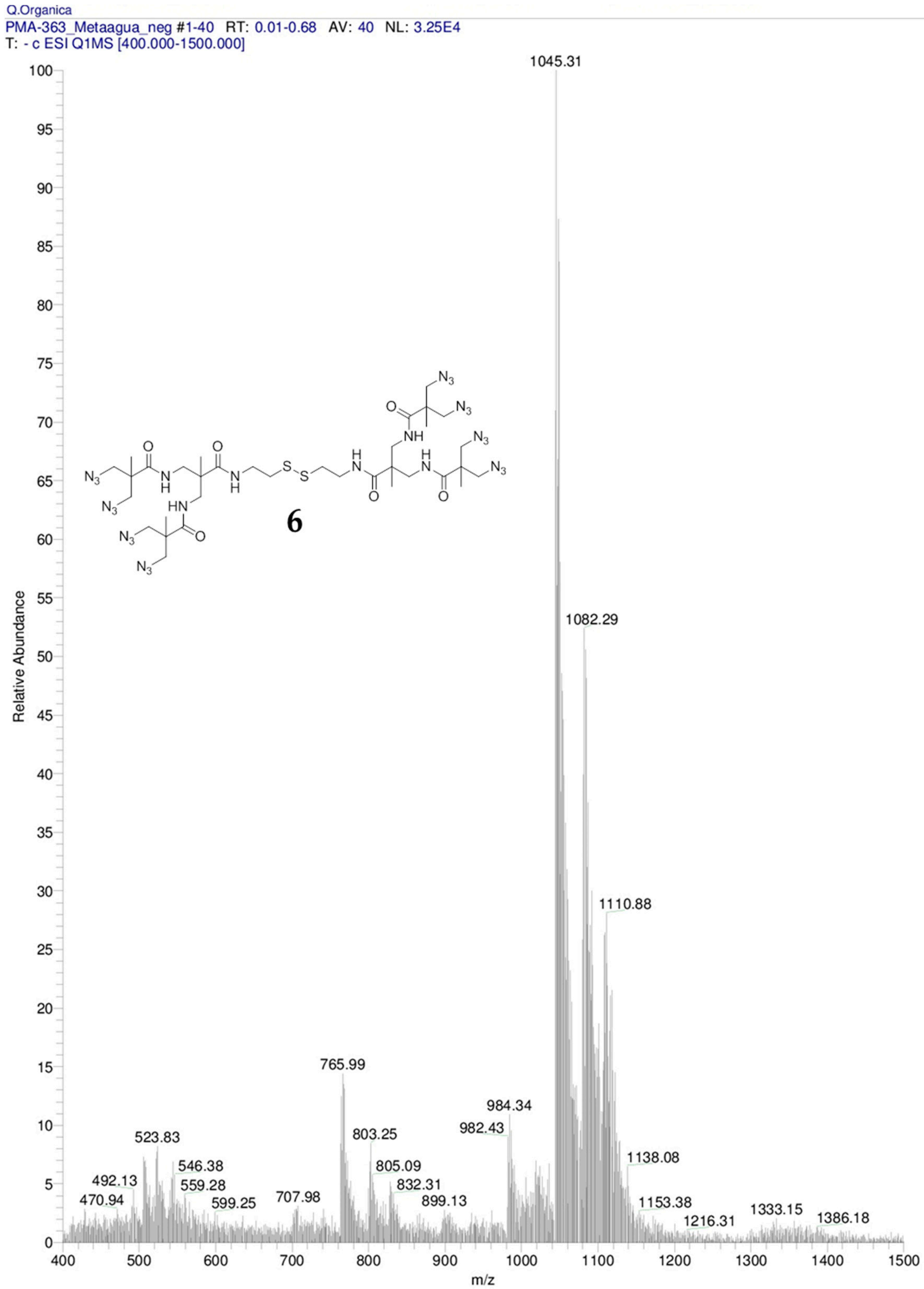

Figure S25. ESI-MS of compound **6**.

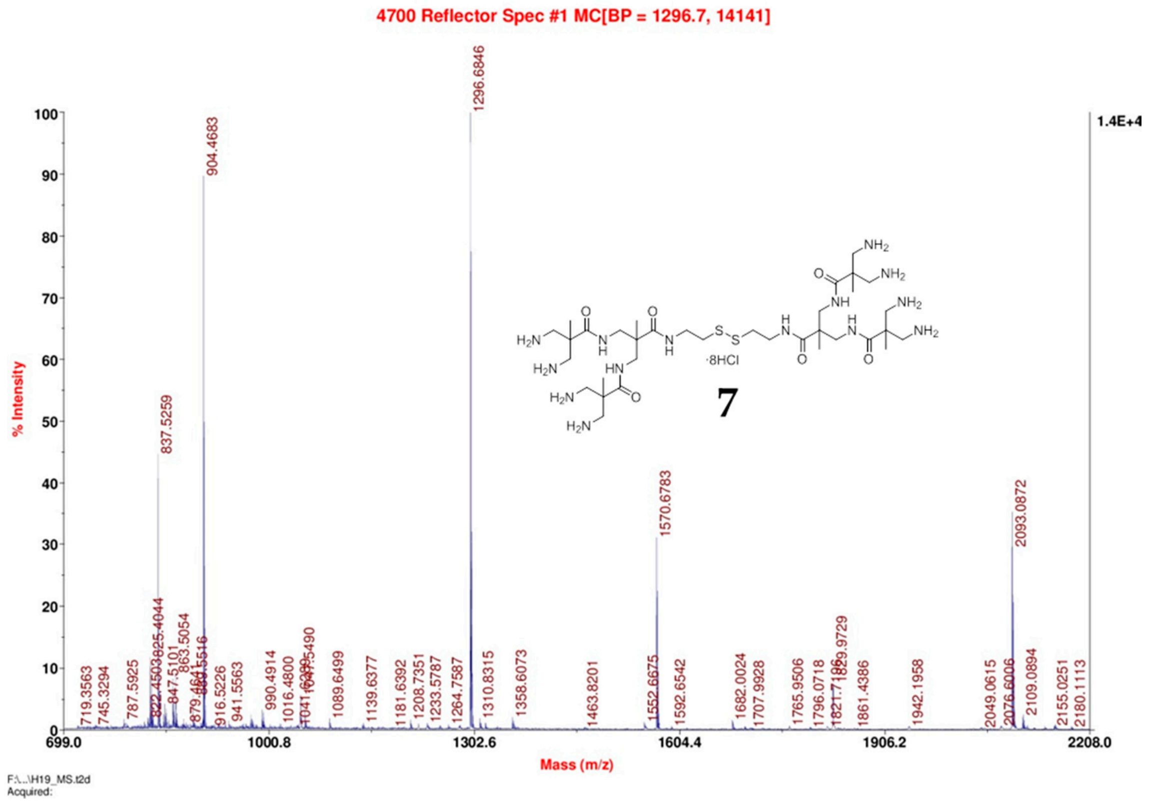

Figure S26. MALDI-TOF-MS of compound **7**.

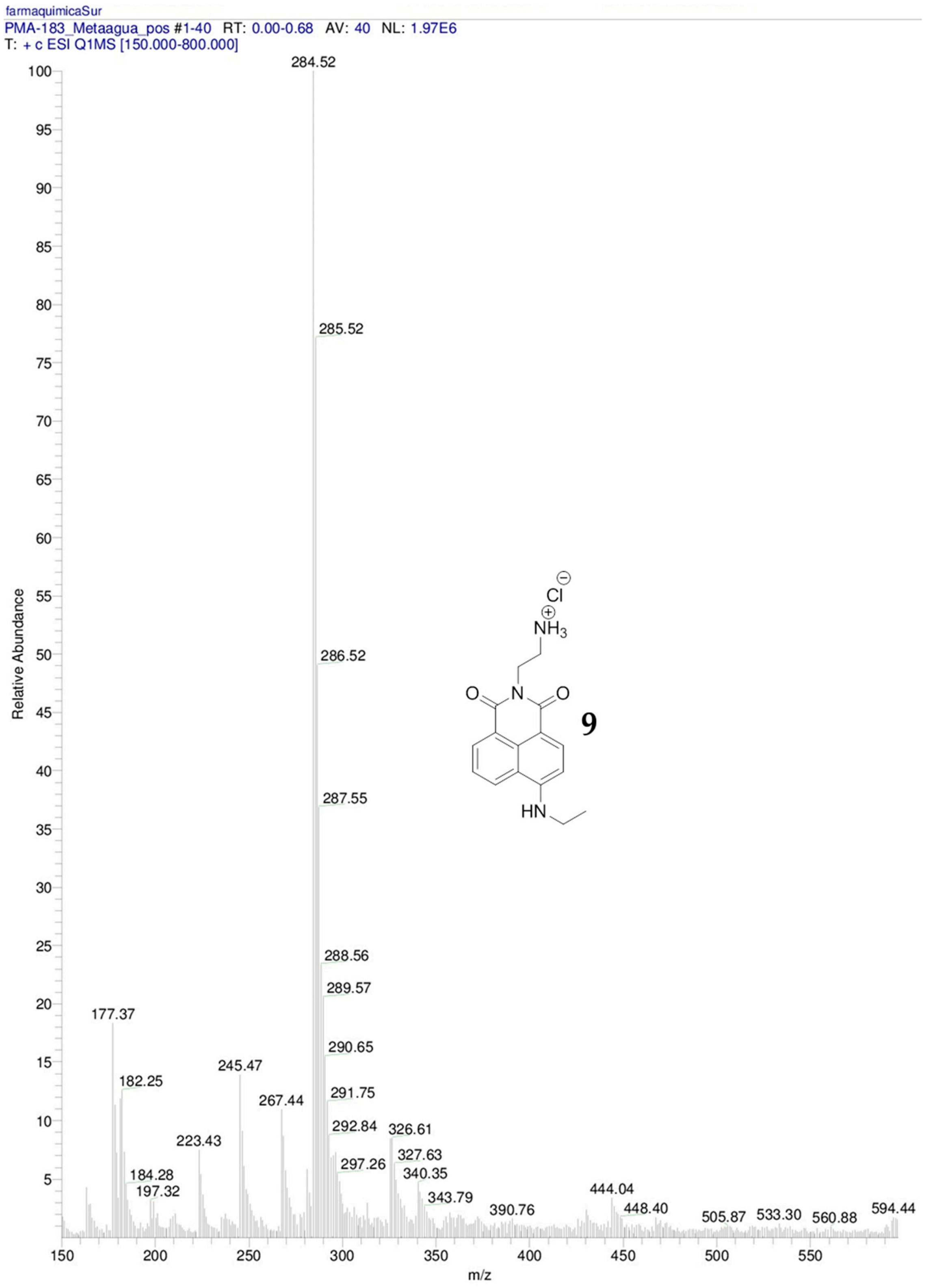

Figure S27. ESI-MS of compound **9**.

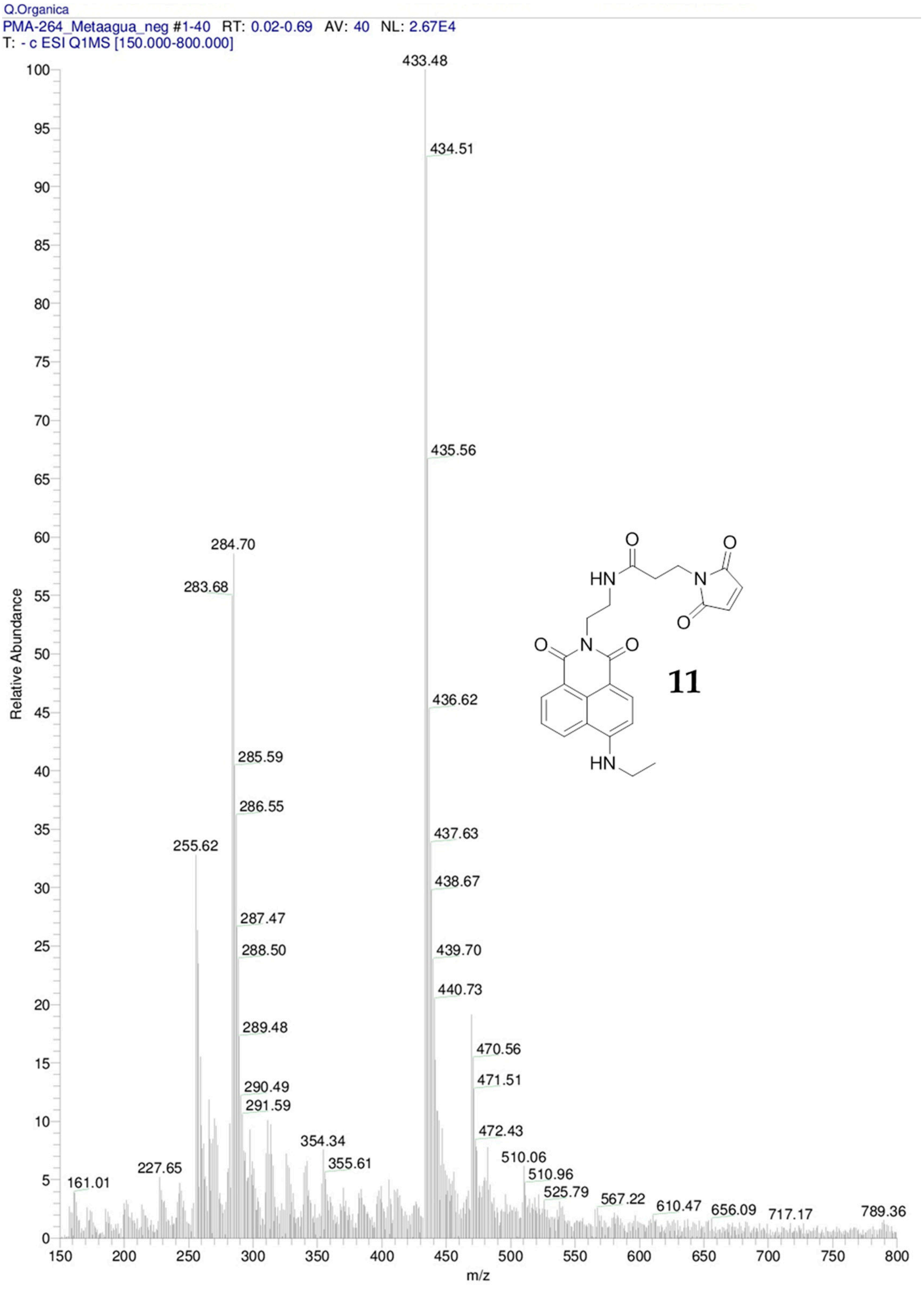

Figure S28. ESI-MS of compound **11**.

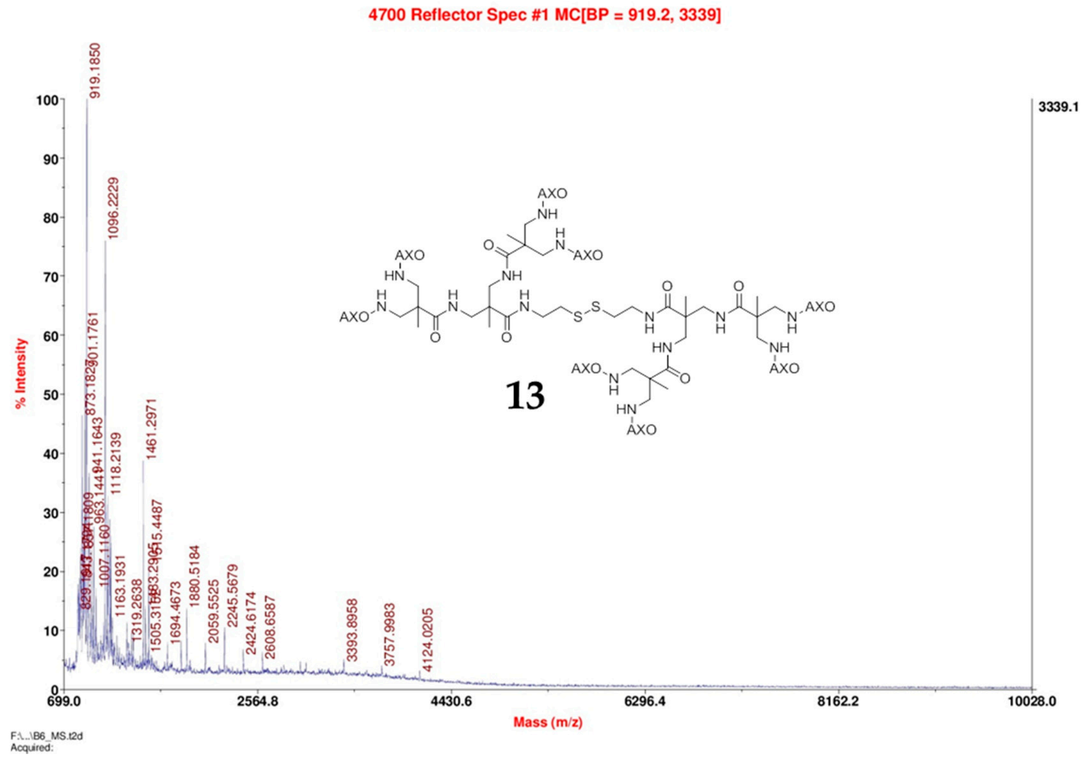

Figure S29. MALDI-TOF-MS of compound **13**.

Absorption and Fluorescence Emission Spectra

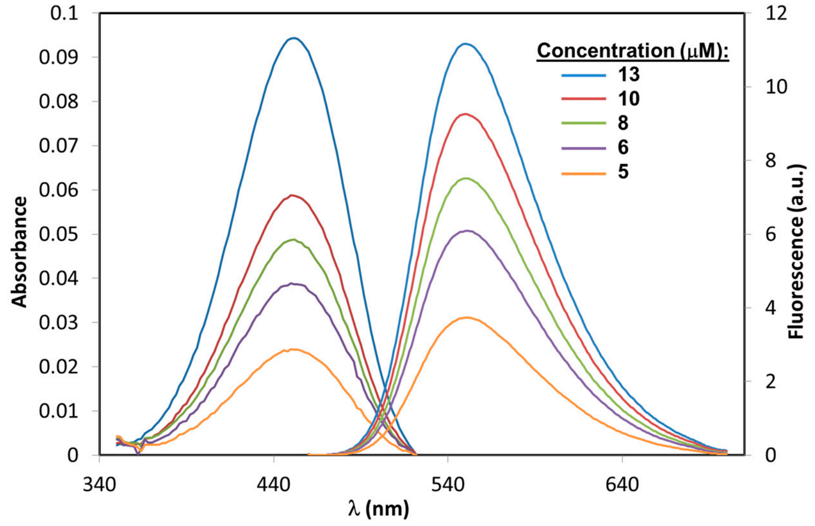

Figure S30. Absorption and fluorescence emission spectra of **1** at different concentration in PBS.

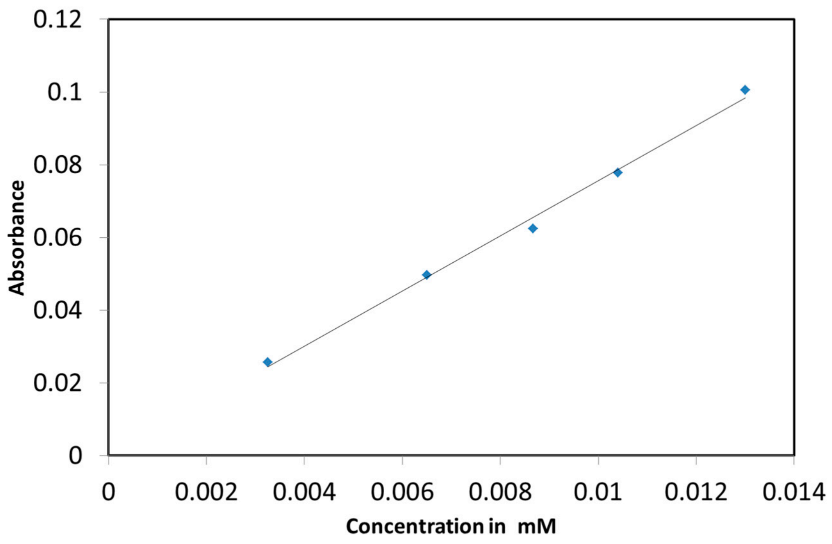

Figure S31. Absorption maximum depending on concentration of **1** in PBS (R^2 = 0.9946).

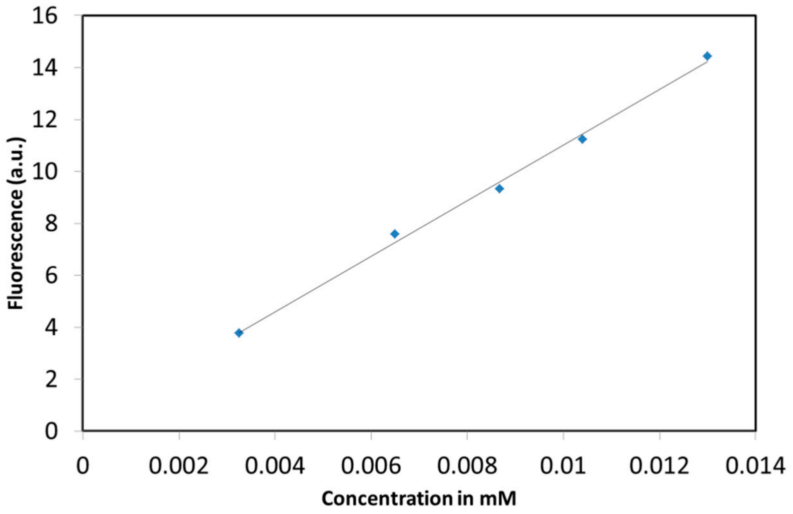

Figure S32. Fluorescence emission maximum depending on concentration of **1** in PBS (R^2 = 0.996).

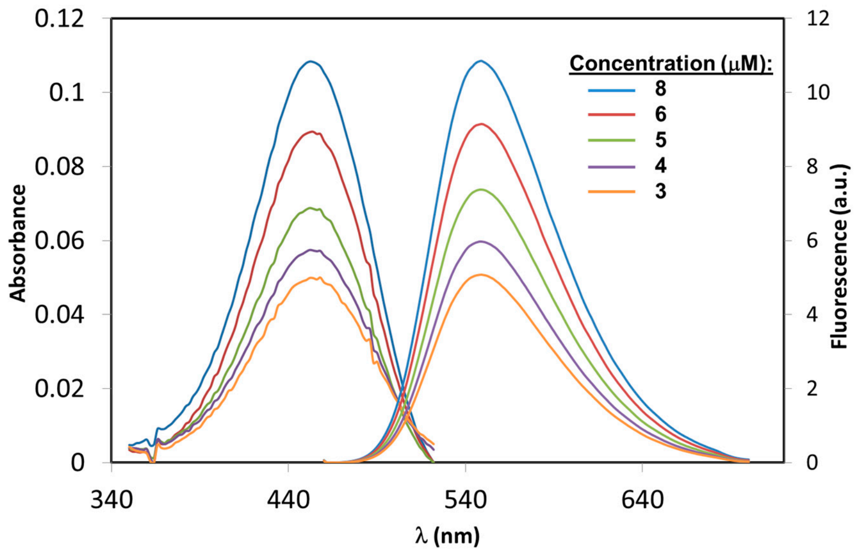

Figure S33. Absorption and fluorescence emission spectra of **2** at different concentration in PBS.

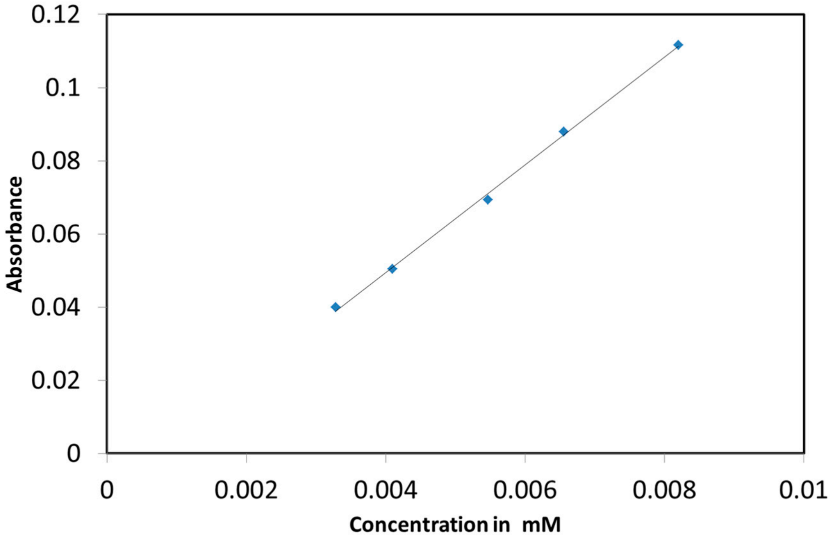

Figure S34. Absorption maximum depending on concentration of **2** in PBS (R^2 = 0.9985).

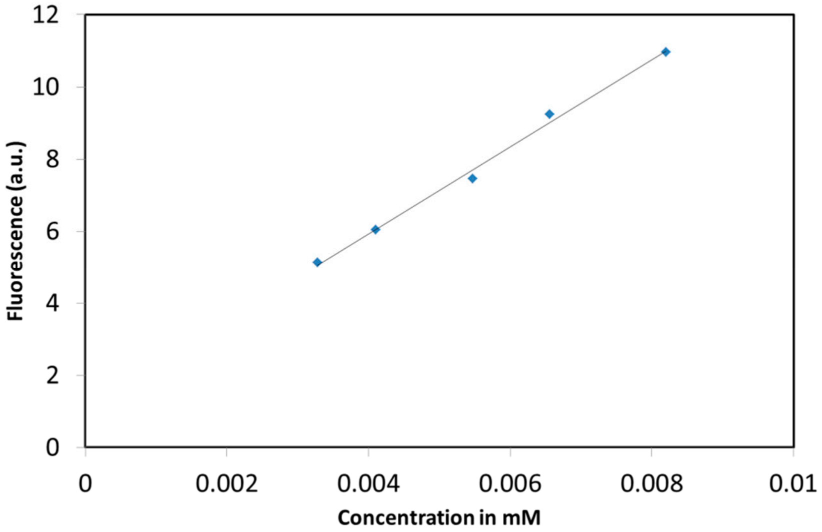

Figure S35. Fluorescence emission maximum depending on concentration of **2** in PBS (R^2 = 0.9948).

Molecular Dynamics Simulations Details

Dendron Building. Dendrons are composed of three different residues: the naphthalimide core with the linker to the BAPAD unit (COR), the repetitive 3,3′-diaminopivaloyl units (REP), and the terminal end 3,3′-diamoniumpivaloyl unit (TAM). For the penicilloyl dendrons the terminal residue was an amoxicilloyl unit (AXM/AXH) (Figure S36). The pK_a values of the amoxicillin are (2.4, 7.4 and 9.6) [1]. Thus, at physiological pH (7.4) the relation between [amoxi-NH_2]/[amoxi-NH_3^+] = 1. Consequently, for the dendrons with amoxicilloyl groups we built them with two different terminal units: a terminal amoxicilloyl group (AXM) and a terminal amino protonated amoxicilloyl group (AXH). Then, equal numbers of terminal AXM and AXH were randomly inserted into the dendrons.

For all of these residues a cap region was defined and for missing bonds, angle, torsions or van der Waals parameters not included in the parm99 force field, the values were transferred from the general AMBER force field (GAFF) [2]. These moiety types were optimized and subjected to conformational analysis using TINKER-Software Tools for Molecular Design [3]. The minimum energy conformation was submitted to PCM(water)/MP2/HF/6-31G(d) basis set calculation using G09 [4]. The restrained potential (RESP) method was used for charge fitting [5]. During charge calculation, total cap atom charge was constrained to zero, and the overall full residue charge was also set to zero

except for the protonated residue (TAM), which was kept at +2. Decapped residues were created using the Antechamber module of Amber 12 [6], and used to build the desired dendrimer generation employing the Dendrimer Building Tool (DBT) [7].

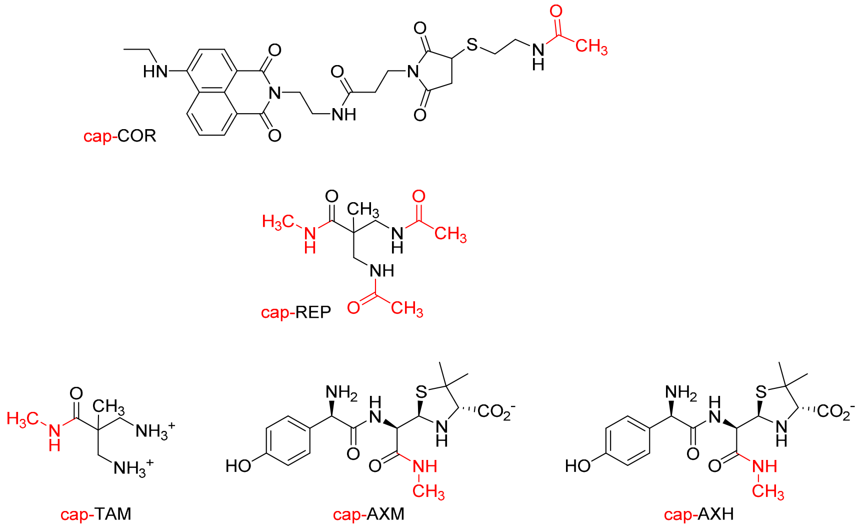

Figure S36. Residue selection for dendrons. Cap atoms are shown in red.

Simulation Details. Full atomistic simulations were performed in water as explicit solvent at neutral pH. We used the AMBER 12 MD software package for all calculations [6]. To preserve overall charge neutrality and represent more realistic conditions for an appropriate number of Cl^- and Na^+ counterions were added, and the molecules solvated, using the TIP3P water model [8], in truncated octahedral cells. In all cases the dimensions of these cells were chosen to provide a minimum 10 Å solvation shell around the dendron structure. The initial properties of dendrons **1** and **2** are collected in Table S1.

Table S1. Initial properties of dendrons and simulation details. N_{den}, N_{Cl^-}, N_{Na^+}, N_{water} and N_{total} are, respectively, the number of dendron atoms, chloride ions, sodium ions, atoms in solvent molecules and the total number of atoms. *V* is the initial octahedral box volume.

Dendron	N_{den}	N_{Cl^-}	N_{Na^+}	N_{water}	N_{total}	V (Å^3)
**1**	123	4	–	6111	6238	74,540
**2**	293	2	4	5916	6215	75,513

Solvated structures were minimized as described previously [7], using six cycles of conjugated gradient minimization. During the initial cycle, dendrimers were kept in their starting conformation using a harmonic constraint with a force constant of 500 kcal/mol-Å^2. This was followed by another five periods of minimization while decreasing the harmonic restraint force constant from 20 kcal/mol-Å^2 to zero in steps of 5 kcal/mol-Å^2.

To allow a slow relaxation of the assembled dendrimer-solvent system, the minimized structure was heated slowly from 0 to 300 K with three steps of 40 ps of MD, the first of them under conditions of constant volume-constant temperature (NVT) and the rest under constant pressure-constant temperature (NPT) conditions. Initially, we applied a weak 20 kcal/mol-Å^2 harmonic constraint to the solute starting structure and slowly decreased it to zero in 5 kcal/mol-Å^2 steps. Finally, we carried out 2 ns of unconstrained MD simulation in NPT ensemble to equilibrate the system at 300 K. To solve the motion equation we used the Verlet leapfrog algorithm [9], with an integration step of 2 fs. Bond lengths involving bonds to hydrogen atoms were constrained using the SHAKE algorithm [10], using a geometrical tolerance of 5×10^{-4} Å.

Finally, starting from the configurations generated by the above procedure, production runs of 40 ns trajectories were performed under an NPT ensemble. Temperature regulation was achieved

using the Berendsen weak coupling method (1 ps time constant for heat bath coupling and 0.5 ps for pressure relaxation time) [11]. The particle-mesh Ewald (PME) algorithm was employed to treat long-range electrostatic interactions [12], with a real space cut off of 9 Å. The same cutoff was used for van der Waals interactions. For the structural analyses (R_g, RDF, *etc.*) the last 1 ns equilibrated trajectory was used. Amber modules *ptraj* and *cpptraj* were used to accomplish these analyses.

Autocorrelation Function

Dendrimer relaxation was determined from the autocorrelation function of the squared radius-of-gyration, $C_{R_g^2}(t)$ which is evaluated from the expression [13]:

$$C_{R_g^2}(t) = \frac{\langle R_g^2(0) R_g^2(t)\rangle - \langle R_g^2\rangle^2}{\langle R_g^4\rangle - \langle R_g^2\rangle^2}$$

The resulting graph is presented in Figure S2. In all cases $C_{R_g^2}(t)$ decays to 0 at time scales shorter than 1 ns, implying that the simulation times are sufficient to sample enough independent configurations in order to average static properties.

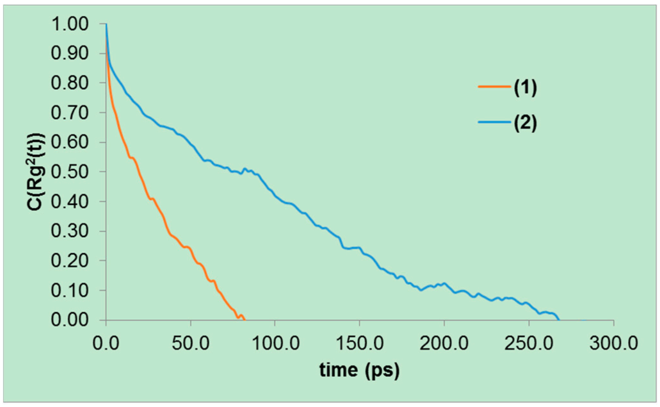

Figure S37. Correlation functions of the squared radius of gyration for dendrons **1** and **2**.

Radius of Gyration

Dendrimer size can be quantified by considering the radius of gyration R_g of the molecular structure, computed as follows:

$$R_g = \left(\frac{\sum_i \|r_i\|^2 m_i}{\sum_i m_i}\right)^{1/2}$$

where m_i is the mass of atom i and r_i the position of atom ***i*** with respect to the molecule's center of mass.

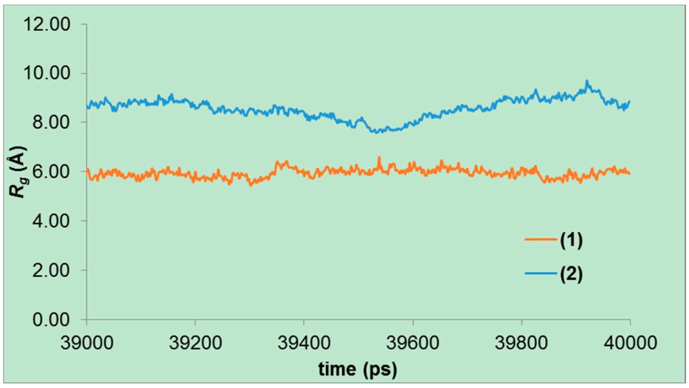

Figure S38. Time evolution of the Radius of Gyration against the last nanosecond for dendrons **1** and **2**.

Radius of the Solvent Accessible Surface Area (SASA)

Assuming a perfect sphere, the Solvent Accessible Surface Area (SASA) is:

$$\mathrm{SASA} = 4\pi(R_{\mathrm{SASA}} + p)^2$$

where R_{SASA} is the radius of the *SASA* and *p* is the radius of the probe. Plotting $(\mathrm{SASA})^{1/2}$ *vs*. *p* show that for larger probe radius, (for these molecules for $p > 4$), $(\mathrm{SASA})^{1/2}$ is linear in *p* with a slope $(4\pi)^{1/2} = 3.54$. The intercept at zero probe radius leads to estimate the area of the molecule as they were spheres.

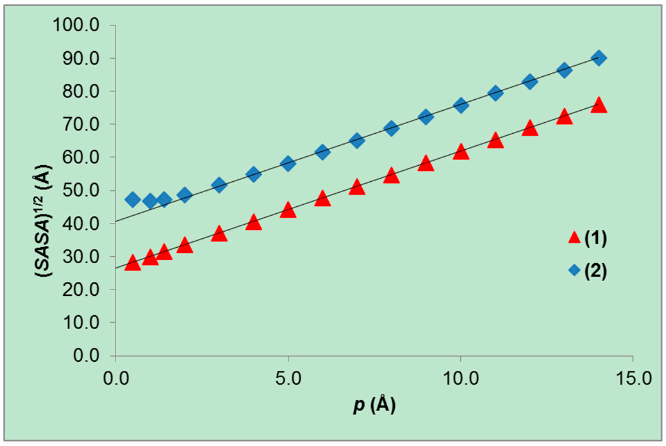

$\mathrm{SASA}^{1/2} = \mathrm{a}(R_{\mathrm{SASA}} + p)$		
**Dendron**	**a**	R_{SASA} **(Å)**
**1**	3.54	7.47
**2**	3.54	11.49

Figure S39. Square root of Solvent Accessible Surface Areas (SASA) as a function of probe radius *p* for dendrons **1** and **2**. A theoretical slope $(4\pi)^{1/2} = 3.54$ was used.

Dendrons Shape

A structure characteristic of dendrons **1** and **2** can be obtained by considering average values of the three principal moments of inertia I_x, I_y, I_z (in descending order). Molecular asphericity was calculated as defined [14]:

$$\delta = 1 - 3\frac{\langle I_2 \rangle}{\langle I_1^2 \rangle}$$

where I_1 and I_2 are the first and second invariants of the radius of gyration tensor: $I_1 = I_x + I_y + I_z$, $I_2 = I_xI_y + I_yI_z + I_xI_z$. This quantity assumes values between 1 (for a linear array of atoms) and 0 (for shapes of high 3D similarity).

Monomer Radial Distribution Functions

Dendrimer conformation can be conveniently expressed through the average radial distribution function $\rho(r)$, which can be defined by counting the number *N(r)* of atoms whose centers of mass are located within the spherical shell of radius *r* and thickness Δr. Hence, integration over *r* yields the total number of atoms as: $N(r) = 4\pi \int_0^\infty r^2\, \rho(r)\, dr$, where *r* is the distance from the dendrimer center of mass.

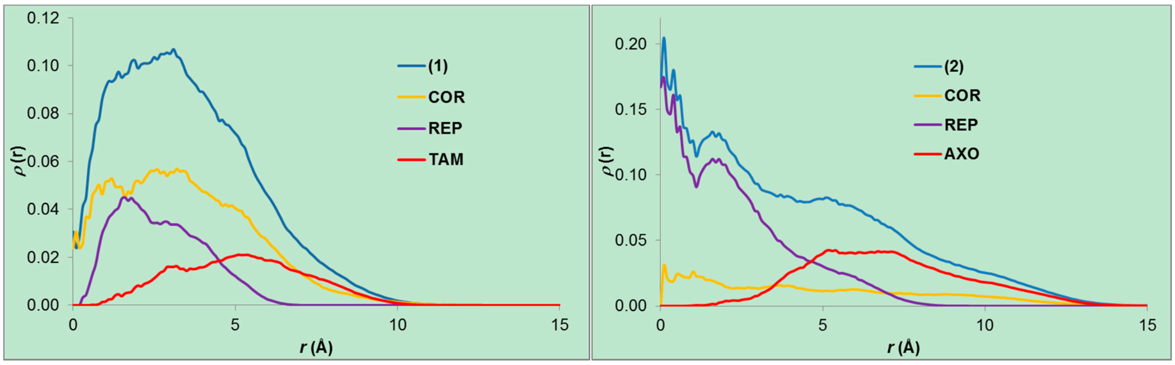

Figure S40. Radial distribution function of dendrons **1** and **2**, with its monomers using dendrimer center of mass as reference. The unit value for $\rho\,(r)$ is expressed in atoms/Å^3.

References

1. Rolinson, G.N. Laboratory evaluation of amoxicillin. *J. Infect. Dis.* **1974**, *129*, S139–S145.
2. Wang, J.; Wolf, R.M.; Caldwell, J.W.; Kollman, P.A.; Case, D.A. Development and testing of a general amber force field. *J. Comput. Chem.* **2004**, *25*, 1157–1174.
3. Ponder, J.W. *TINKER—Software Tools for Molecular Design*; (http://dasher.wustl.edu/tinker/, accessed March 2016).
4. Frisch, M.J.; Trucks, G.W.; Schlegel, H.B.; Scuseria, G.E.; Robb, M.A.; Cheeseman, J.R.; Scalmani, G.; Barone, V.; Mennucci, B.; Petersson, G.A.; *et al. Gaussian 09, Revision A.1*; Gaussian, Inc.: Wallingford, CT, USA, 2009.
5. Bayly, C.I.; Cieplak, P.; Cornell, W.; Kollman, P.A. A well-behaved electrostatic potential based method using charge restraints for deriving atomic charges: The RESP model. *J. Phys. Chem.* **1993**, *97*, 10269–10280.
6. Case, D.A.; Darden, T.A.; Cheatham, T.E., III; Simmerling, C.L.; Wang, J.; Duke, R.E.; Luo, R.; Walker, R.C.; Zhang, W.; Merz, K.M.; *et al. AMBER 12*; AMBER; University of California: San Francisco, CA, USA, 2012.
7. Maingi, V.; Jain, V.; Bharatam, P.V.; Maiti, P.K. Dendrimer building toolkit: Model building and characterization of various dendrimer architectures. *J. Comput. Chem.* **2012**, *33*, 1997–2011.
8. Jorgensen, W.L.; Chandrasekhar, J.; Madura, J.D.; Impey, R.W.; Klein, M.L. Comparison of simple potential functions for simulating liquid water. *J. Chem. Phys.* **1983**, *79*, 926–935.
9. Verlet, L. Computer "experiments" on classical fluids. I. Thermodynamical properties of lennard-jones molecules. *Phys. Rev.* **1967**, *159*, 98–103.
10. Ryckaert, J.-P.; Ciccotti, G.; Berendsen, H.J.C. Numerical integration of the cartesian equations of motion of a system with constraints: Molecular dynamics of n-alkanes. *J. Comput. Phys.* **1977**, *23*, 327–341.
11. Berendsen, H.J.C.; Postma, J.P.M.; Gunsteren, W.F.; van; DiNola, A.; Haak, J.R. Molecular dynamics with coupling to an external bath. *J. Chem. Phys.* **1984**, *81*, 3684–3690.
12. Darden, T.; York, D.; Pedersen, L. Particle mesh Ewald: An N⊗log(N) method for Ewald sums in large systems. *J. Chem. Phys.* **1993**, *98*, 10089–10092.
13. Dalakoglou, G.K.; Karatasos, K.; Lyulin, S.V.; Lyulin, A.V. Effects of topology and size on statics and dynamics of complexes of hyperbranched polymers with linear polyelectrolytes. *J. Chem. Phys.* **2007**, *127*, 214903.
14. Rudnick, J.; Gaspari, G. The asphericity of random walks. *J. Phys. Math. Gen.* **1986**, *19*, L191.

© 2016 by the authors; licensee MDPI, Basel, Switzerland. This article is an open access article distributed under the terms and conditions of the Creative Commons by Attribution (CC-BY) license (http://creativecommons.org/licenses/by/4.0/).
